# Supplementary material for: Access and Continuity: A Multidisciplinary Education Workshop to Teach Patient-Centered Medical Home (PCMH) Principles
Source: MedEdPORTAL. 2020 Oct 7;16:10974. doi: 10.15766/mep_2374-8265.10974 (PMC7549388; doi:10.15766/mep_2374-8265.10974)
Supplement: Supplementary file 1 — Prework.docxReflective Activity Prompt Slides.pptxReflective Activity Signs for Walls.docxFaculty Guide.docxSlide Presentation.pptxEvaluation Sheet.docx [file mep_2374-8265.10974-s001.zip › A. Prework.docx]

**PCMH Principles Workshop: Access and Continuity**

**Prework Activities**

1. Call your primary care provider and try to make an appointment. Note down when the next available sick visit is at that office, when the next available physical appointment is with your provider and a covering provider if available sooner. If you don’t have a primary care provider, call your local urgent care center and see if they take your insurance and what the copay is, and go to the Northwell website and find out the wait time and copay for your nearest emergency department.

| **With your PCP** | **With your PCP** | **With a covering provider at your PCP’s office** | **Urgent Care** | **Local ER wait time** |
| --- | --- | --- | --- | --- |
| Next available sick visit (“bad sore throat”) |  |  |  |  |
| Annual Physical |  |  |  |  |

1. Please share any thoughts or notes below:

**Pre-reading for PCMH Workshop**

**Introduction to the Patient-Centered Medical Home**

The concept of the “patient-centered medical home” (PCMH) is still fluid and somewhat variable, being defined by a number of different regulating bodies, both public and private. As the PCMH becomes more important in the payment model of our health care system, its definition is becoming more refined and specific in order to have consistent underlying principles for what is in fact a very flexible model for health care delivery. The following is one description of the core principles of PCMH.

**AHRQ Principles and Concepts of the PCMH^^[[1]](#footnote-1)^^**

The Agency for Healthcare Research and Quality (AHRQ) is a government agency within the larger Department of Health and Human Services.

According to the AHRQ and the Patient-Centered Primary Care Collaborative (which based their definition on AHRQ’s), the PCMH is built around **five core principles and functions**:

- **Comprehensive care.** The PCMH is oriented toward the “whole person” and is responsible for addressing all the patient’s physical and mental acute, chronic, and preventive health care needs. This involves the direct provision of the appropriate care when possible or arranging for other qualified professionals (such as specialists) to provide care when necessary. Care within the primary care setting is delivered by a team rather than a single clinician, so professionals with different skill sets are available to meet the patient’s needs.
- **Patient-centered approach.** The PCMH provides care that is relationship based and tailored to best meet each patient’s needs, values, culture, and preferences. Each patient has the opportunity to build ongoing, trusting relationships with a team of health care professionals. Clinicians seek to engage patients in their health care; provide the support, education, and information they need to make informed health care decisions; and recognize them as important members of the care team. PCMH clinicians and health care professionals use their cultural competence to treat patients with dignity, respect, and compassion, and they seek to meet patients where they are so that care is delivered in the way that best suits the patient’s needs.
- **Coordinated care.** All of a patient’s health care is coordinated by the PCMH, including care received in hospitals, from specialists (including mental and behavioral health specialists), and through community or home-based services and supports. Coordination of care may be facilitated by patient registries, use of health information technology (such as electronic health records), and other methods. To ensure that care is properly coordinated, the PCMH strives to build strong communication with patients and among all members of a patient’s care team. The goal of coordination is greater efficiency through avoidance of duplication of services, synchronization of services so that they have a maximum impact, and ensuring connection of patients to needed services.
- **Accessibility of services.** To ensure that patients are able to access care when they need it, the PCMH offers short wait times for urgent care, enhanced hours, and around-the-clock access to the care team via telephone or electronic methods (email, patient portal, etc.). Care teams also seek out and respond to patient preferences regarding access and communication (e.g., whether patients prefer to communicate via email or telephone, and what language they prefer to use when getting care).
- **Quality and safety.** To achieve optimal patient health outcomes and the highest quality of care, the PCMH is committed to quality improvement (QI), performance improvement, patient satisfaction, and population health management. Practices use evidence-based medicine and decision support tools to guide shared decision-making and use patient registries to track the health status of their entire patient panel. Practices use data-driven QI methodologies to continuously monitor performance in a variety of care areas. Patients are engaged in QI processes and involved in practice decision making to ensure that care is provided in accordance with patient wants and needs.

These principles closely align with the core values of primary care, which are defined by the Institute of Medicine as providing integrated, accessible health care services to meet the majority of personal health care needs in a sustained partnership with patients in the context of family and community.”

1. Higgins T.C., Schottenfeld L, Crosson J. Primary Care Practice Facilitation Curriculum (Module 25). AHRQ Publication No. 15-0060-EF, Rockville, MD: Agency for Healthcare Research and Quality; September 2015. Accessed at <https://pcmh.ahrq.gov/sites/default/files/attachments/pcpf-module-25-pcmh-principles.pdf> on August 2, 2016. [↑](#footnote-ref-1)
