# Supplementary material for: Access and Continuity: A Multidisciplinary Education Workshop to Teach Patient-Centered Medical Home (PCMH) Principles
Source: MedEdPORTAL. 2020 Oct 7;16:10974. doi: 10.15766/mep_2374-8265.10974 (PMC7549388; doi:10.15766/mep_2374-8265.10974)
Supplement: Supplementary file 1 — Prework.docxReflective Activity Prompt Slides.pptxReflective Activity Signs for Walls.docxFaculty Guide.docxSlide Presentation.pptxEvaluation Sheet.docx [file mep_2374-8265.10974-s001.zip › B. Reflective Activity Prompt Slides.pptx]

## Slide 1
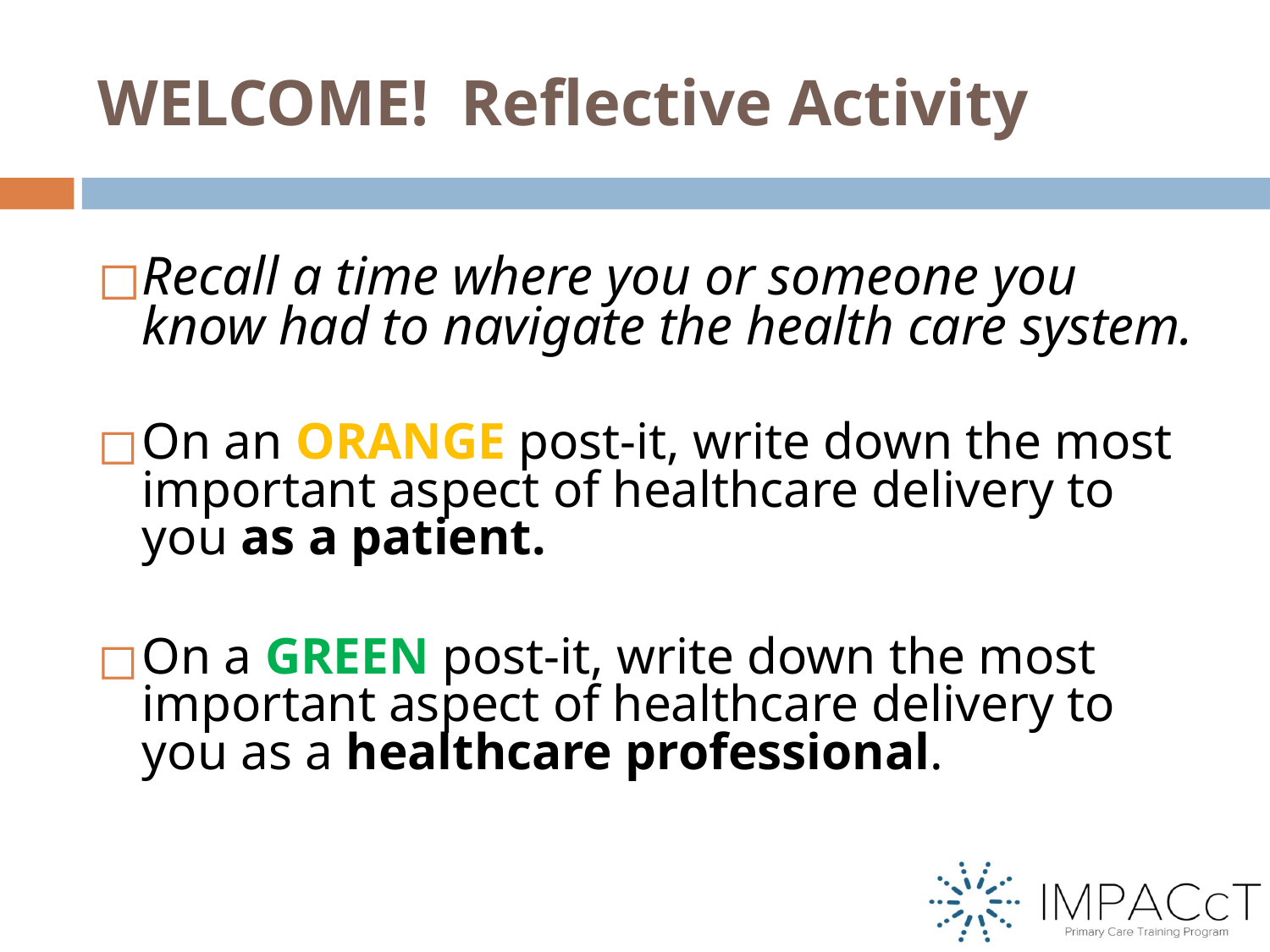

# WELCOME! Reflective Activity
Recall a time where you or someone you know had to navigate the health care system.
On an ORANGE post-it, write down the most important aspect of healthcare delivery to you as a patient.
On a GREEN post-it, write down the most important aspect of healthcare delivery to you as a healthcare professional.

## Slide 2
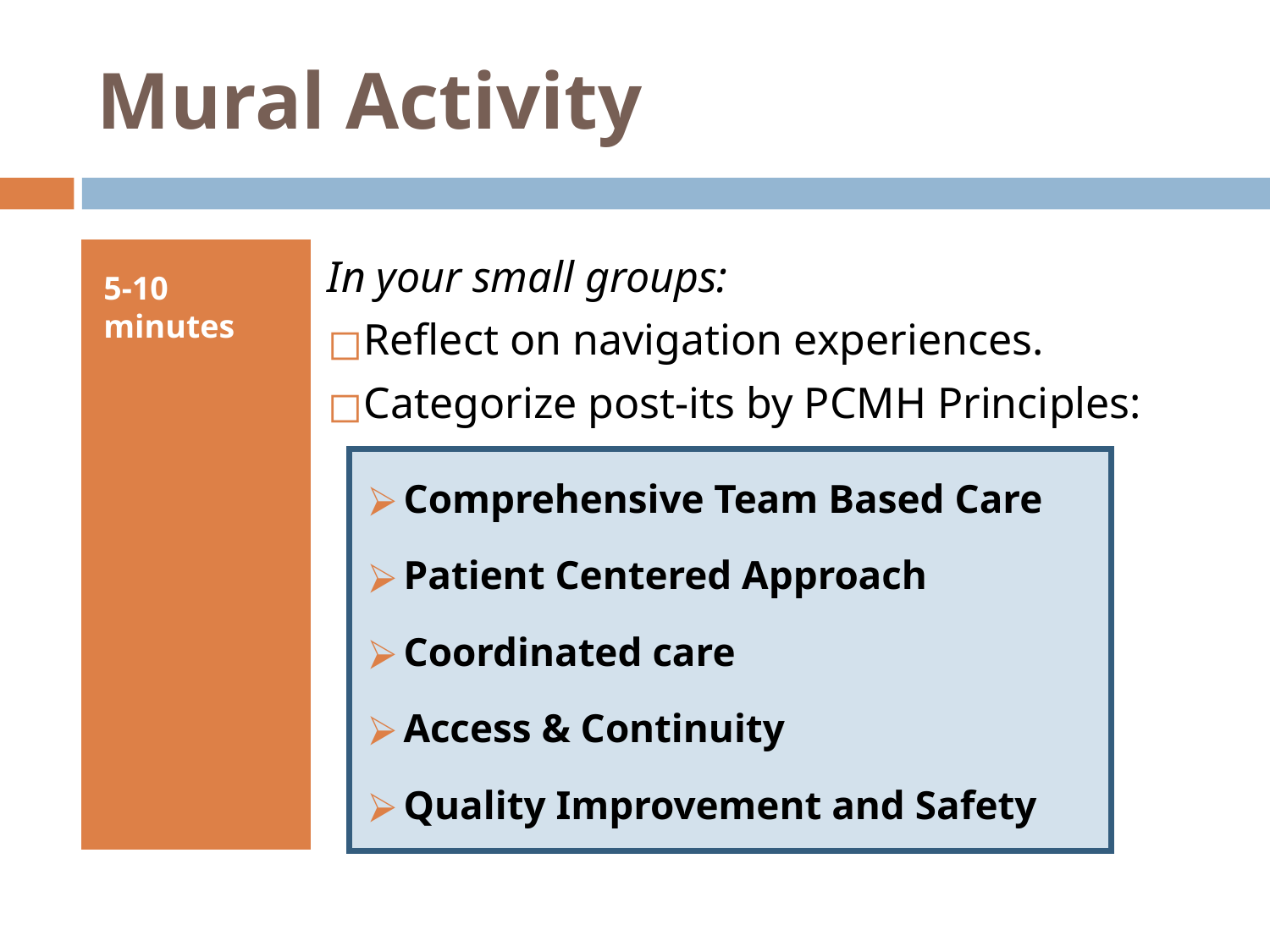

# Mural Activity
5-10 minutes
In your small groups:
Reflect on navigation experiences.
Categorize post-its by PCMH Principles:
Comprehensive Team Based Care
Patient Centered Approach
Coordinated care
Access & Continuity
Quality Improvement and Safety
