# Supplementary material for: Access and Continuity: A Multidisciplinary Education Workshop to Teach Patient-Centered Medical Home (PCMH) Principles
Source: MedEdPORTAL. 2020 Oct 7;16:10974. doi: 10.15766/mep_2374-8265.10974 (PMC7549388; doi:10.15766/mep_2374-8265.10974)
Supplement: Supplementary file 1 — Prework.docxReflective Activity Prompt Slides.pptxReflective Activity Signs for Walls.docxFaculty Guide.docxSlide Presentation.pptxEvaluation Sheet.docx [file mep_2374-8265.10974-s001.zip › C. Reflective Activity Signs for Walls.docx]

**Comprehensive**

**Team-Based Care**

**Patient Centered Approach**

**Coordinated care**

**Access &**

**Continuity**

**Quality Improvement and Safety**
