# Supplementary material for: Access and Continuity: A Multidisciplinary Education Workshop to Teach Patient-Centered Medical Home (PCMH) Principles
Source: MedEdPORTAL. 2020 Oct 7;16:10974. doi: 10.15766/mep_2374-8265.10974 (PMC7549388; doi:10.15766/mep_2374-8265.10974)
Supplement: Supplementary file 1 — Prework.docxReflective Activity Prompt Slides.pptxReflective Activity Signs for Walls.docxFaculty Guide.docxSlide Presentation.pptxEvaluation Sheet.docx [file mep_2374-8265.10974-s001.zip › D. Faculty Guide.docx]

**PCMH Principles Workshop: Access and Continuity**

**FACILITATOR GUIDE**

**Learning Objectives**

1. Compare and contrast patient- and provider-centered care.
2. Identify the components of a Patient Centered Medical Home (PCMH).
3. Experience the ways patients access care in an ambulatory continuity practice.
4. Brainstorm aspects of an access policy for a resident based practice, including appointment availability and standards for access to obtain clinical advice.
5. Appreciate the interpersonal skills needed to work within a PCMH model of healthcare delivery.
6. Identify ways to enhance continuity of care in a resident practice.
7. Articulate the tension between access and continuity in the delivery of primary care.

**Attachments**

Appendix A: Prework

Appendix B: Reflective Activity Prompt Slides

Appendix C: Reflective Activity Signs for Walls

Appendix E: Slide Presentation

- PCMH Didactic: Slides 1-13
- Continuity: Slides 14-25
- Access: Slides 26-37

Appendix F: Evaluation Sheet

**Prework (Appendix A)**

Students will have pre-reading assigned on PCMH principles.

They will also have the following two activities:

1. Call their PCP or urgent care clinic to make a sick visit or appointment for a routine physical
2. Look up ED or Urgent Care wait times

**Session Outline**

1. Reflective Activity (40 minutes), Appendices B and C
2. Break (10 minutes)
3. PCMH Didactic Session (20 minutes) Appendix E, Slides 1-13
4. Continuity Activity (40 minutes) Appendix E, Slides 14-25
5. Break (10 minutes)
6. Access Activity (40 minutes) Appendix E Slides 26-37
7. Evaluation (10 minutes) Appendix F

Total: 3 hours, 10 minutes

**Module 1: Reflective Activity (40 minutes), Appendices B and C**

**Summary:**

1. Students prepare their index cards and categorize at table while settling into session/eating (15 min)
2. Recall a time you, a family member, or a friend had to navigate the healthcare landscape and what that was like for you.
3. Discussion on principles and values (25 minutes)
   1. Small group discussion (10 minutes)
   2. Put up index cards (5 min)
   3. Large group discussion (10 minutes)

**Instructions:**

Learners will be given orange and green post-it notes and instructed as written below (they also have these instructions in their packet). While they get settled into the session, instruct them to complete the post-its.

**Student Instructions**

*You have been given 2 post-it notes. On the orange post-it, write down the most important aspect of healthcare delivery to you* ***as a patient****.*

*On the green post-it, write down the most important aspect of healthcare delivery to you* ***as a healthcare professional****.*

*Organize the post-its into each of the different PCMH principle categories as labeled on sheets of paper at your tables.*

On your own set of post-it notes, write down the same as asked of the students. Your post-its will be the seed for the mural/mind map that will be created in this activity. Some ideas for important aspects of healthcare delivery:

**As a patient:**

- Clear communication

- Easy to get appointments
- Low cost

**As a healthcare provider:**

- Team support/teamwork
- Adequate IT infrastructure (e.g. EMR access, etc.)
- Community resources to point patients to
- Guidelines/protocols to drive decision-making

Table leaders will facilitate small group discussion at tables (5-10 minutes) about the principles of PCMH that will be listed out on tables, as well as those from the pre-reading, and whether or not the values identified on post-its by the participants fit well into the principles of PCMH. Some ideas for discussion questions are listed below.

When everyone is done with their small group discussion, ask a volunteer from each table to come up and place their groups’ post-its on the wall within the categories of PCMH that they discussed onto the collective mural.

Open for large group debrief (10-15 min). Especially discuss the following: Recall a time you, a family member, or a friend had to navigate the healthcare landscape and what that was like for you.

**Suggested Discussion Questions:**

1. Share your reflections on what is important to patients in navigating the healthcare system.
2. Compare and contrast the values that we identified on our own cards to the principles of PCMH. How are they similar? How are they different?
3. What are some differences between the values identified from a patient perspective vs. from a healthcare provider perspective?
4. How can the values of providers and patients be better aligned?
5. What are your thoughts on PCMH principles after completing this activity?
6. What was a value that a peer identified that resonates with you and why?
7. Do your particular roles, experiences, and knowledge affect the values that you identified compared to those of others?

***Break (10 minutes)***

**Module 2: PCMH Didactic Session (20 minutes) Appendix E, Slides 1-13**

**Module 3: Continuity Activity (40 minutes) Appendix E, Slides 14-25**

**Summary:**

1. Didactic on definitions of continuity (10 minutes)
2. Small group discussion improving continuity at local clinic practice (15 minutes), slide 25
3. Report out to large group (15 minutes)

**Instructions:**

Learners should be in multidisciplinary groups at tables of 6-7 people led by residents and instructed to go through the discussion questions for this activity. They will then report out a brief summary of their discussion to the large group.

**Discussion Questions**

1. What does continuity mean to you?
2. What does continuity mean in your clinic?
3. What are some strengths in continuity in your clinic?
4. What are some areas for improvement of continuity in your clinic?
5. Choose 1-2 ideas to report out to the larger group.

***Break 10 minutes***

**Module 4: Access Activity (40 minutes) Appendix E Slides 26-37**

**Summary:**

1. Revisit pre-work activity in large group (10 min) Slide 27
2. Review slides on Access (measurement, solutions, etc) 10 min
3. Small Group Discussion questions (20min), Slide 35

**Large group:** Review overall prework data on wait for physical and sick visit appointments with PCP and covering provider. Review data on urgent care and ED wait times. (Slide 27)

**Discussion Questions:**

- What did you experience while doing this activity?
- What is important to you in terms of access of care?

**Small Group Discussion (Slide 35):**

**Discussion Questions:**

1. Think about wait times your colleagues experienced in the prework activity. How long is reasonable to wait for:

-Annual physical appointment

-Acute visit appointment for “bad sore throat” or a similar condition

-Call back after phone call

-Notifications after labs

-Discussion of illness over the weekend

1. What might be some complications in making an appointment (e.g. language barriers, hearing difficulties, phone problems, availability of health providers not matching work schedule)?
2. How does access data from your local clinic compare to difficulties your colleagues identified?
3. Using some of the models presented earlier in didactics, is access a problem in your clinic:
   1. Brainstorm a few possibilities to improve one of the access difficulties a member of your team encountered that you could conceivably work on in your local clinic. Vote on one idea to explore further.
   2. What barriers will we encounter in enacting this access improvement initiative? How may we overcome these barriers?

Small groups report out to the large group emphasizing effort and reward for the access improvement activity.

**Evaluation: Appendix F**
