# Supplementary material for: Access and Continuity: A Multidisciplinary Education Workshop to Teach Patient-Centered Medical Home (PCMH) Principles
Source: MedEdPORTAL. 2020 Oct 7;16:10974. doi: 10.15766/mep_2374-8265.10974 (PMC7549388; doi:10.15766/mep_2374-8265.10974)
Supplement: Supplementary file 1 — Prework.docxReflective Activity Prompt Slides.pptxReflective Activity Signs for Walls.docxFaculty Guide.docxSlide Presentation.pptxEvaluation Sheet.docx [file mep_2374-8265.10974-s001.zip › E. Slide Presentation.pptx]

## Slide 1
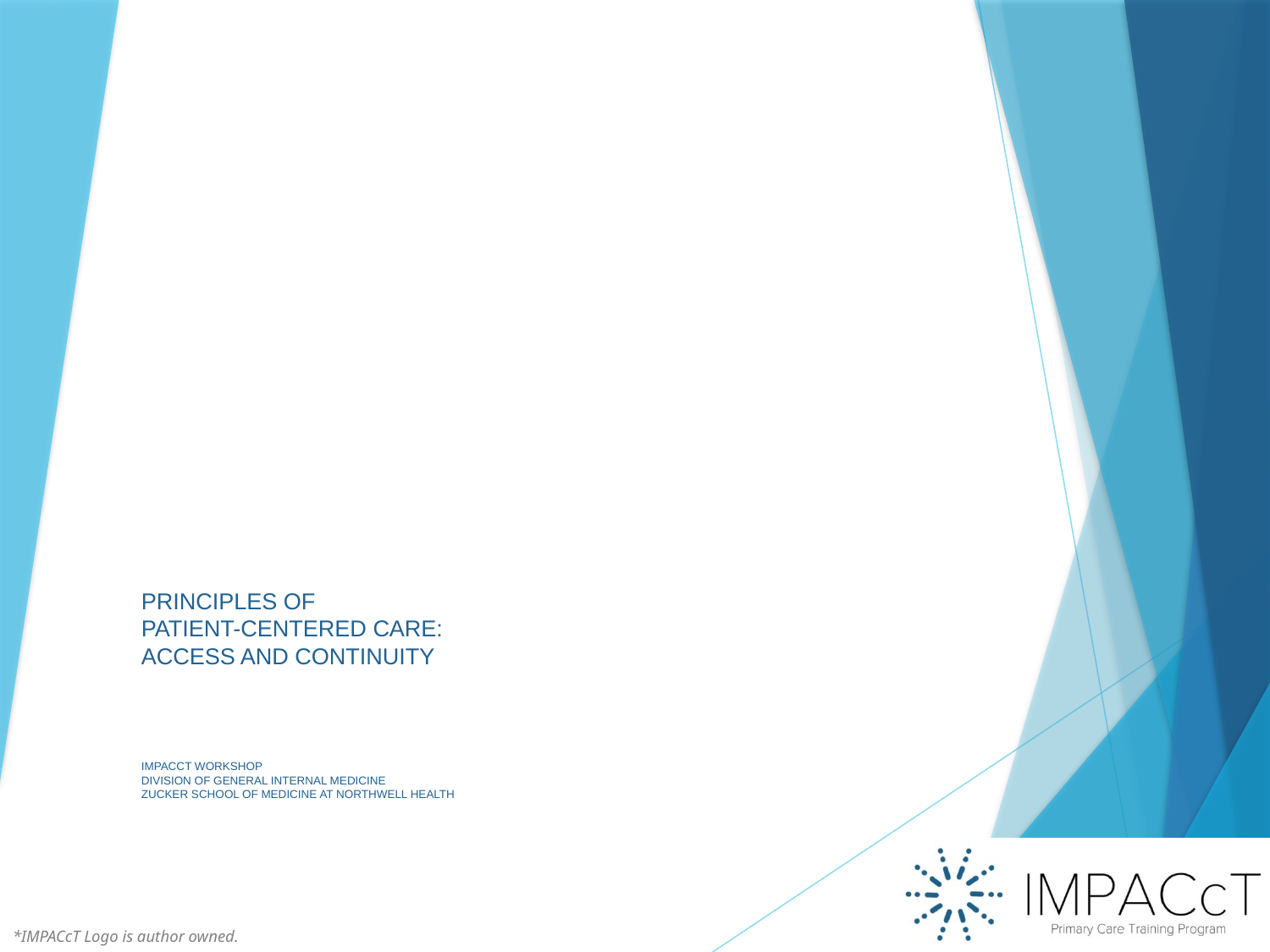

# PRINCIPLES OF PATIENT-CENTERED CARE: ACCESS AND CONTINUITY IMPACCT WORKSHOP DIVISION OF GENERAL INTERNAL MEDICINE ZUCKER SCHOOL OF MEDICINE AT NORTHWELL HEALTH
1
*IMPACcT Logo is author owned.

## Slide 2
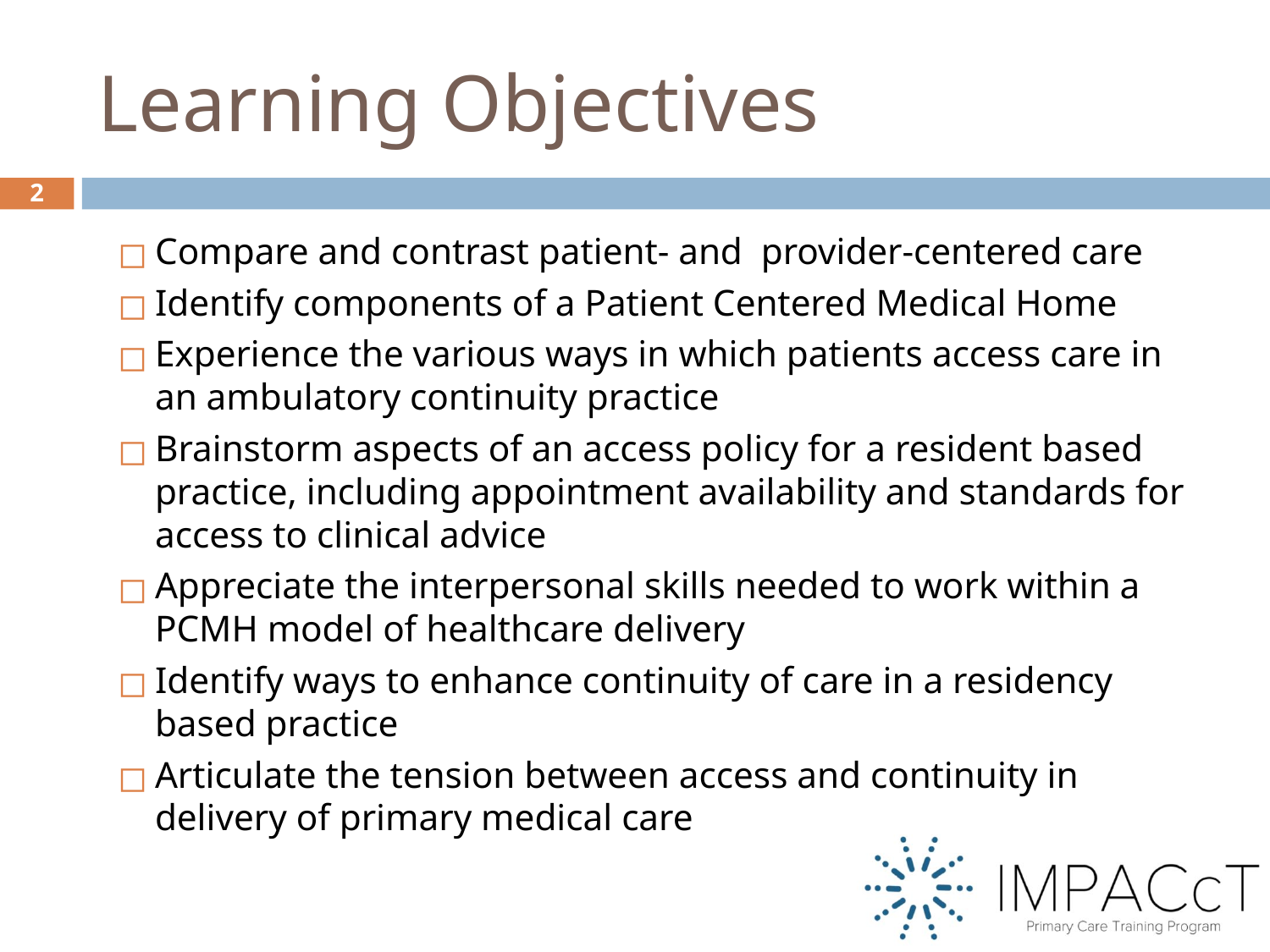

# Learning Objectives
2
Compare and contrast patient- and provider-centered care
Identify components of a Patient Centered Medical Home
Experience the various ways in which patients access care in an ambulatory continuity practice
Brainstorm aspects of an access policy for a resident based practice, including appointment availability and standards for access to clinical advice
Appreciate the interpersonal skills needed to work within a PCMH model of healthcare delivery
Identify ways to enhance continuity of care in a residency based practice
Articulate the tension between access and continuity in delivery of primary medical care

## Slide 3
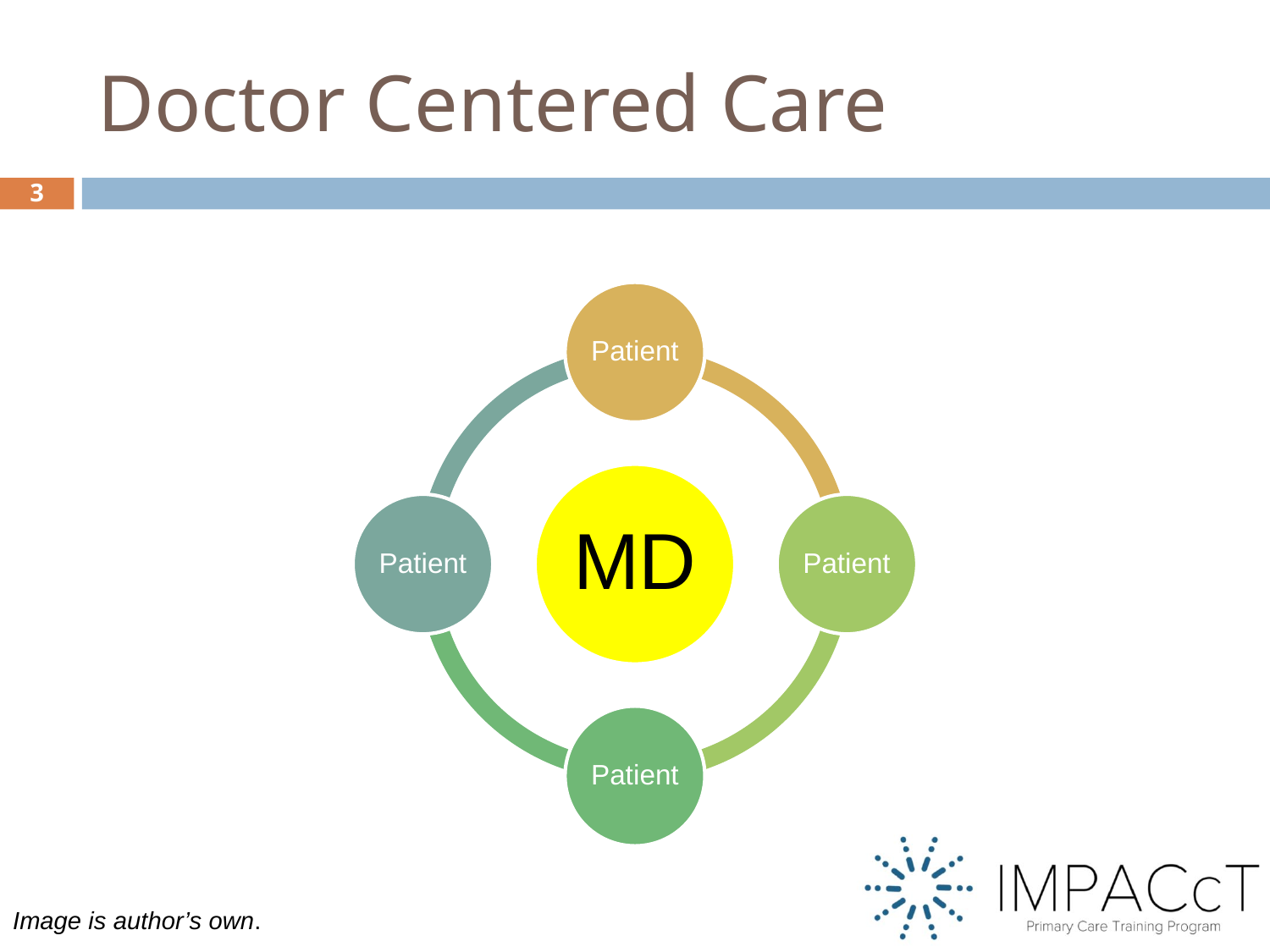

# Doctor Centered Care
3
Image is author’s own.

## Slide 4
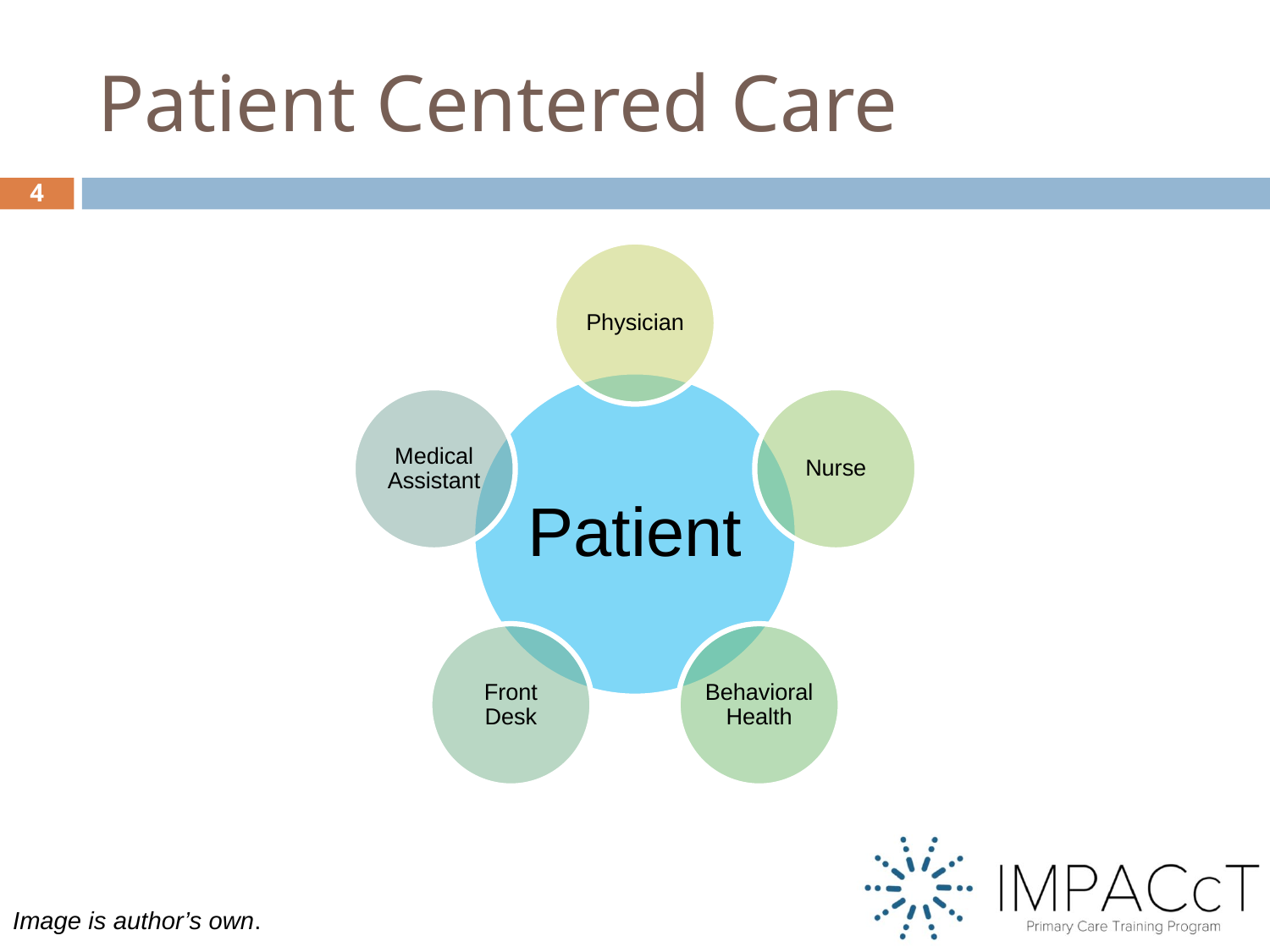

# Patient Centered Care
4
Image is author’s own.

## Slide 5
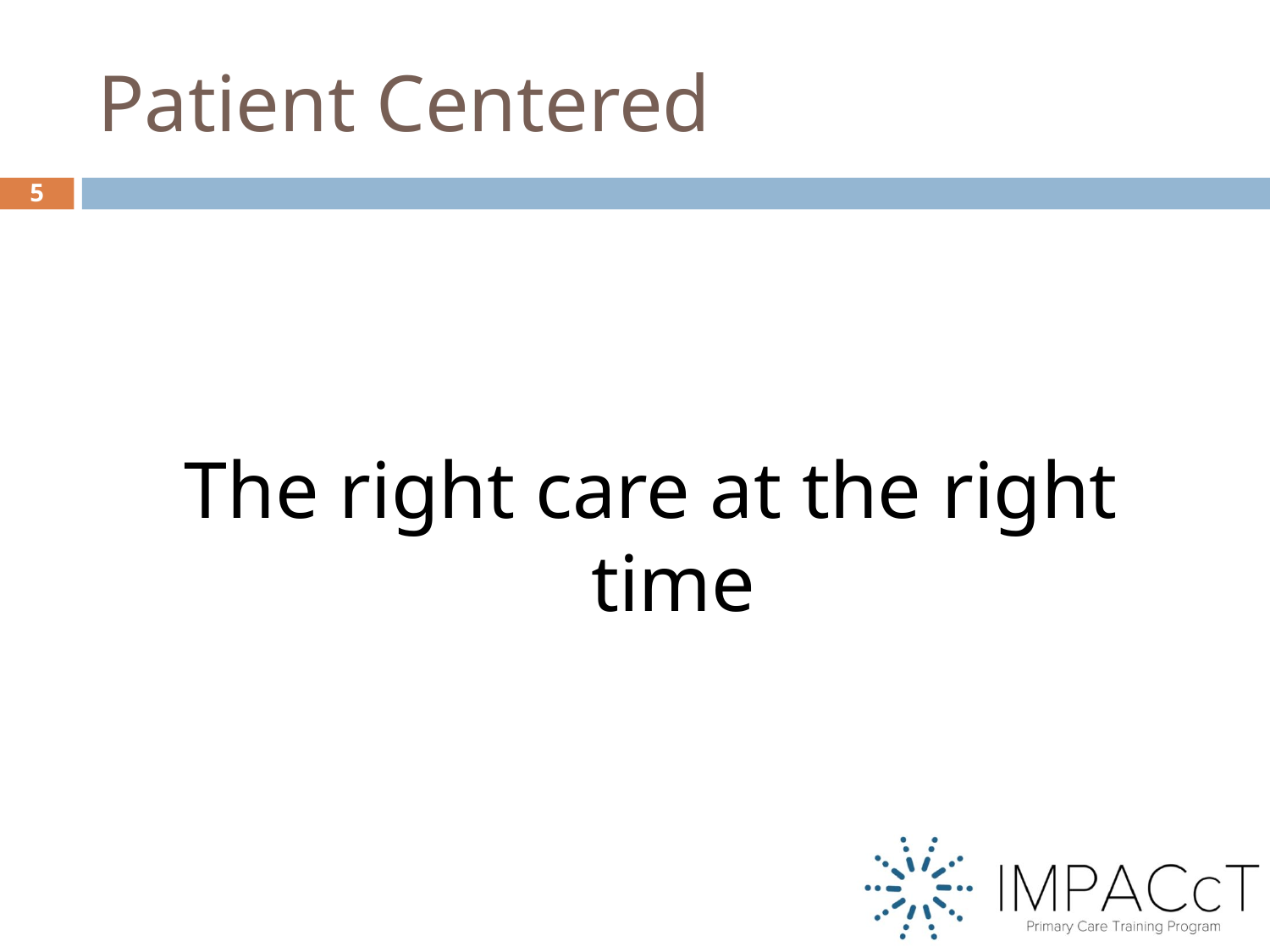

# Patient Centered
5
The right care at the right time

## Slide 6
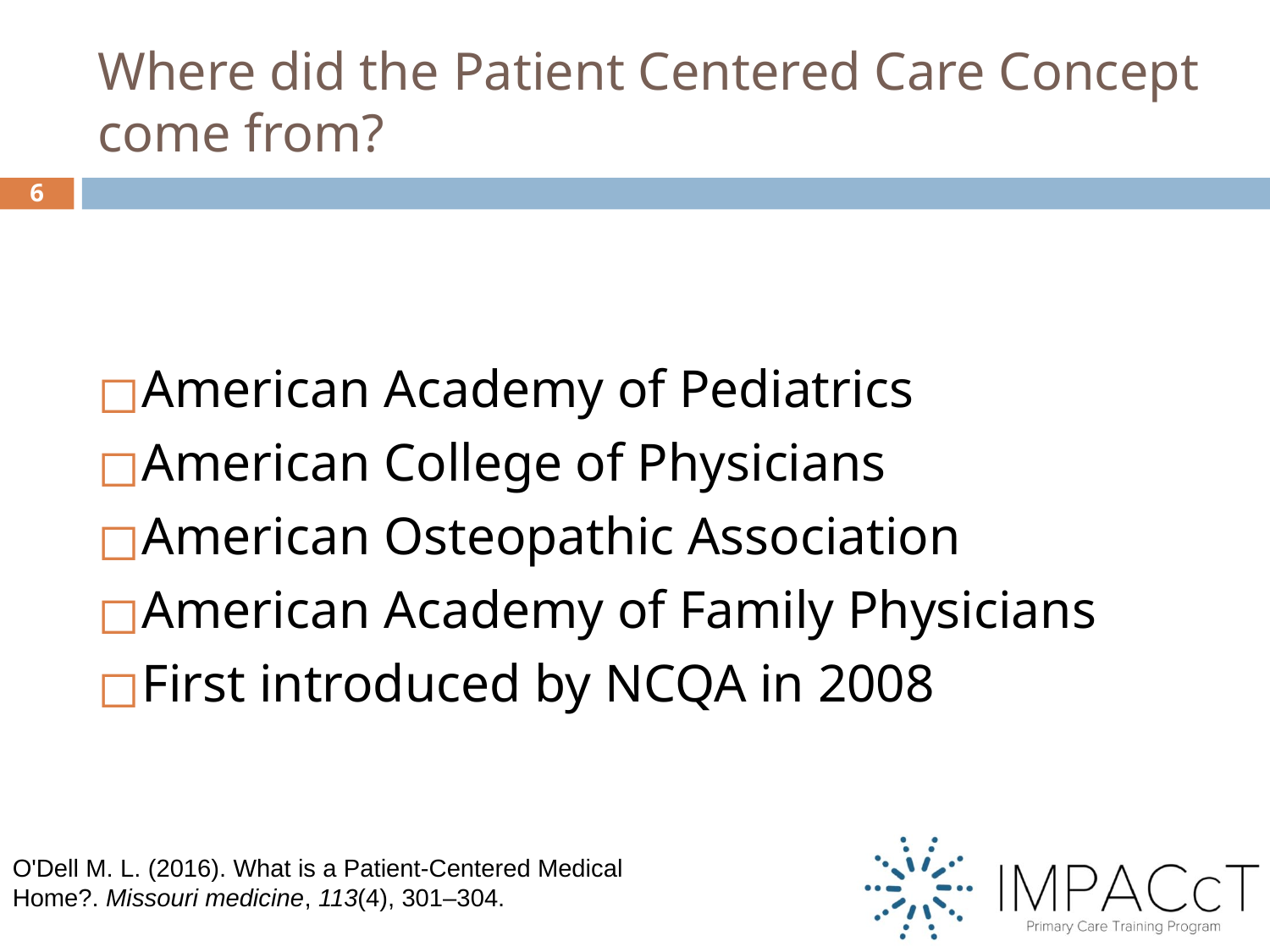

# Where did the Patient Centered Care Concept come from?
6
American Academy of Pediatrics
American College of Physicians
American Osteopathic Association
American Academy of Family Physicians
First introduced by NCQA in 2008
O'Dell M. L. (2016). What is a Patient-Centered Medical Home?. Missouri medicine, 113(4), 301–304.

## Slide 7
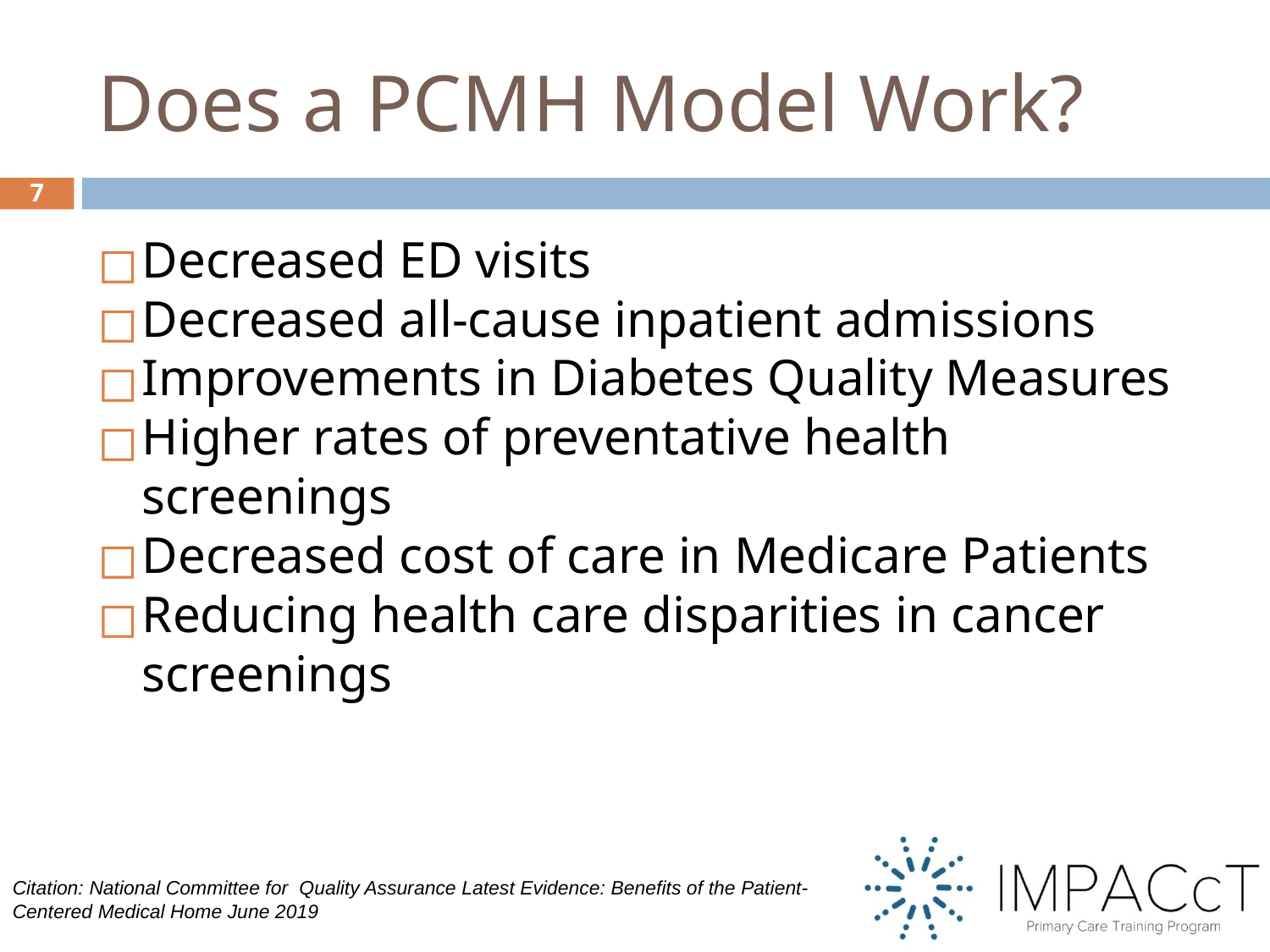

# Does a PCMH Model Work?
7
Decreased ED visits
Decreased all-cause inpatient admissions
Improvements in Diabetes Quality Measures
Higher rates of preventative health screenings
Decreased cost of care in Medicare Patients
Reducing health care disparities in cancer screenings
Citation: National Committee for Quality Assurance Latest Evidence: Benefits of the Patient-Centered Medical Home June 2019

## Slide 8
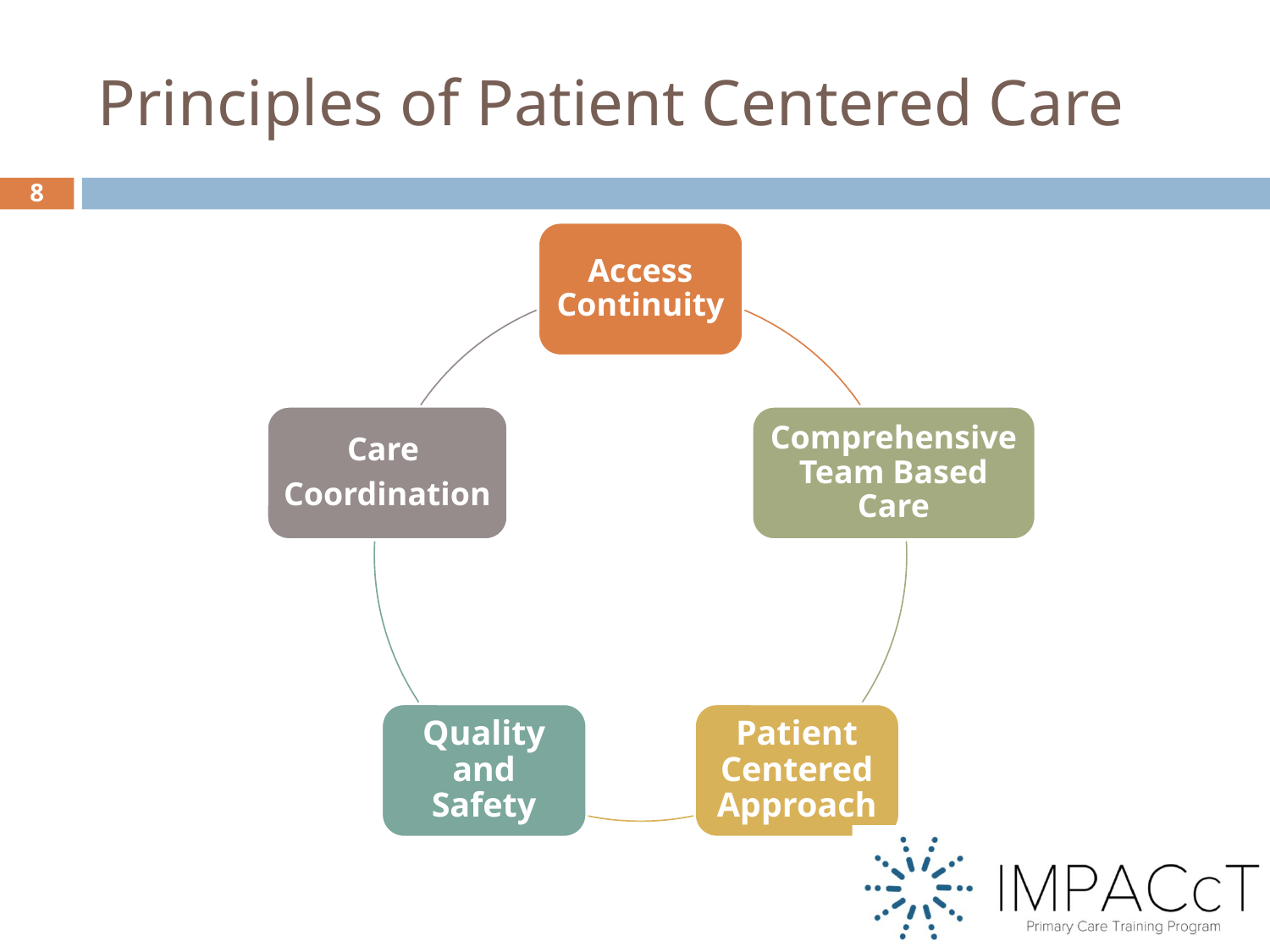

# Principles of Patient Centered Care
8
Access Continuity
Care
Coordination
Comprehensive Team Based Care
Quality and Safety
Patient Centered Approach

## Slide 9
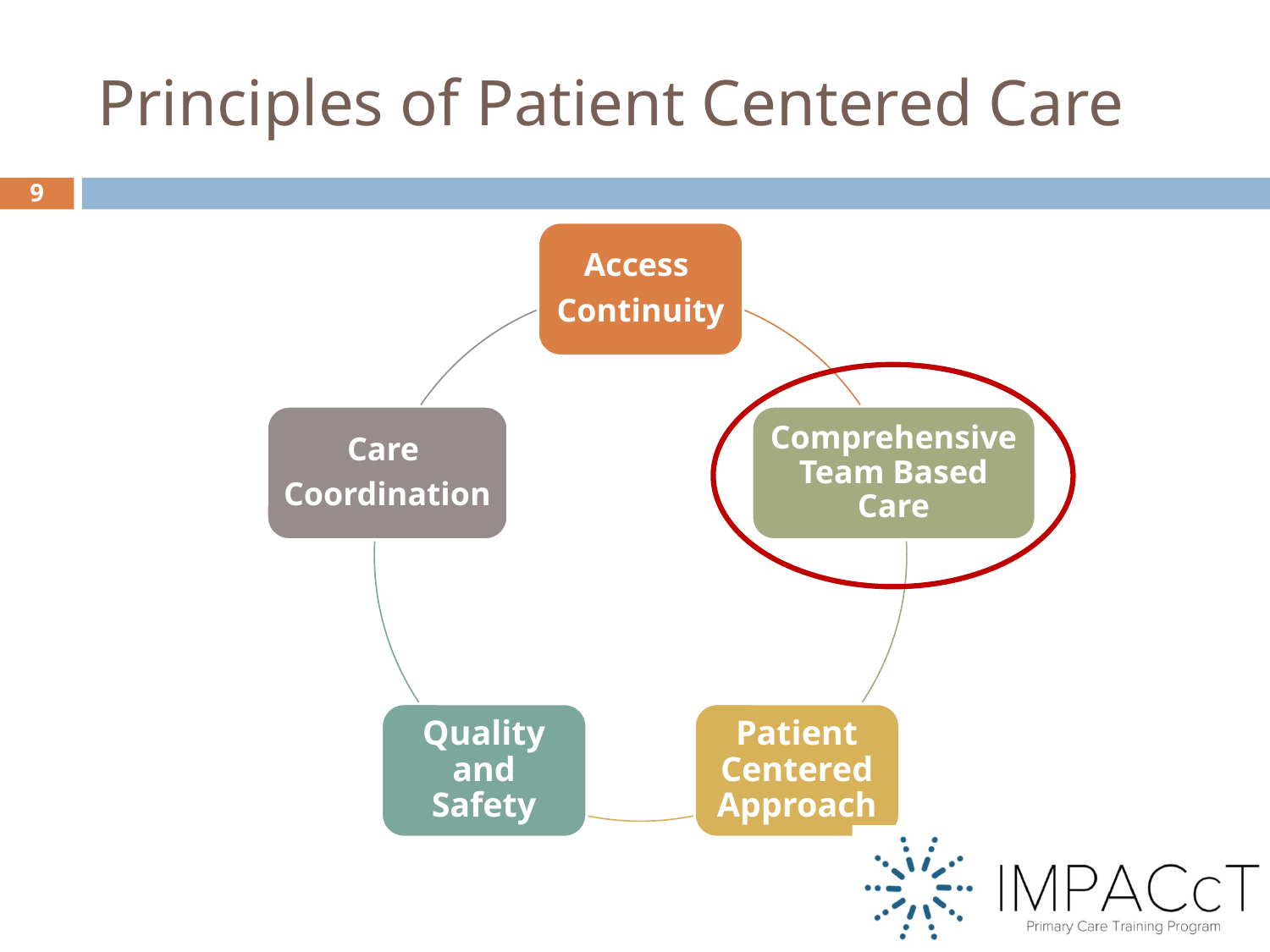

# Principles of Patient Centered Care
9
Access
Continuity
Care
Coordination
Comprehensive Team Based Care
Quality and Safety
Patient Centered Approach

## Slide 10
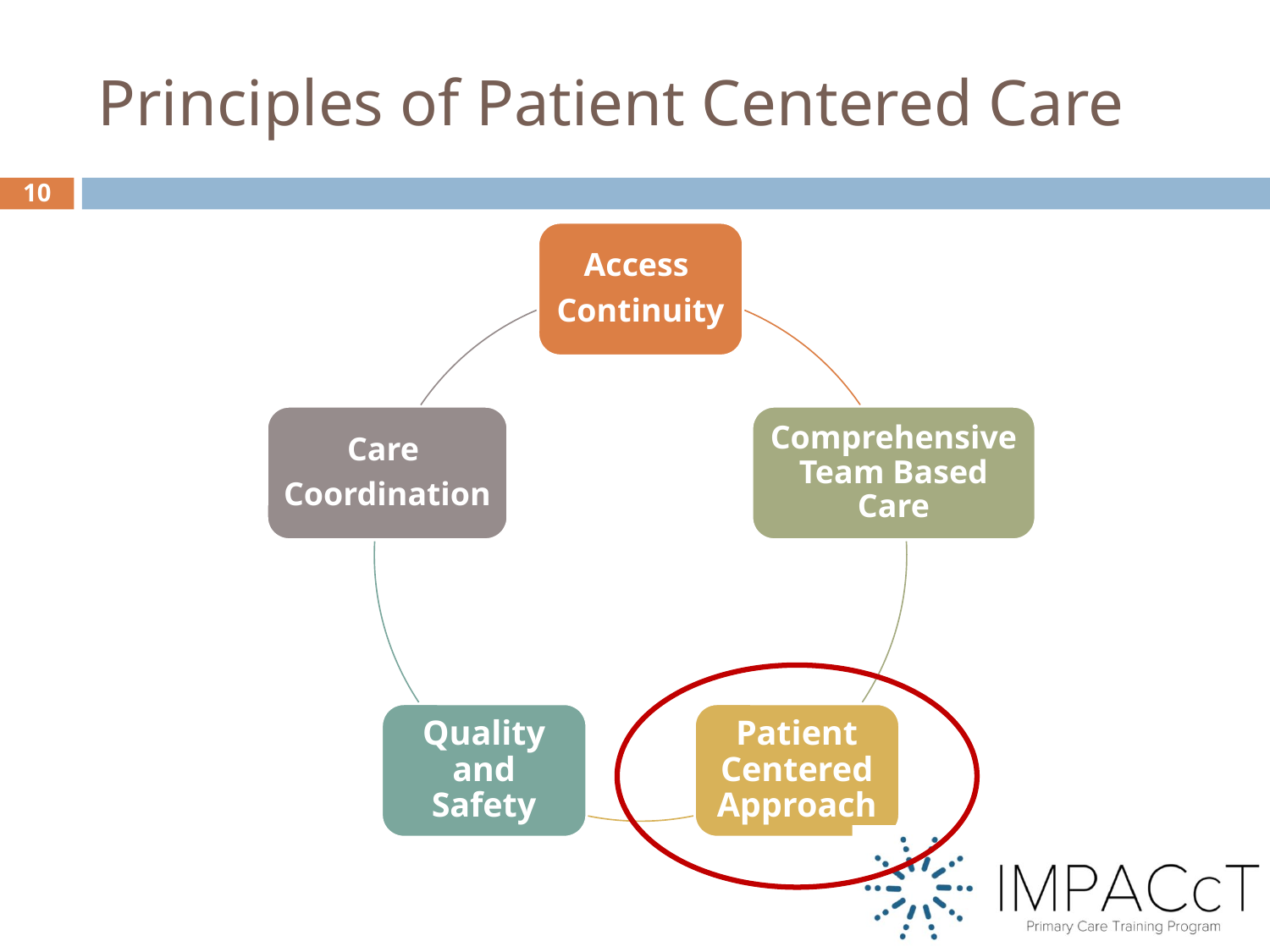

# Principles of Patient Centered Care
10
Access
Continuity
Care
Coordination
Comprehensive Team Based Care
Quality and Safety
Patient Centered Approach

## Slide 11
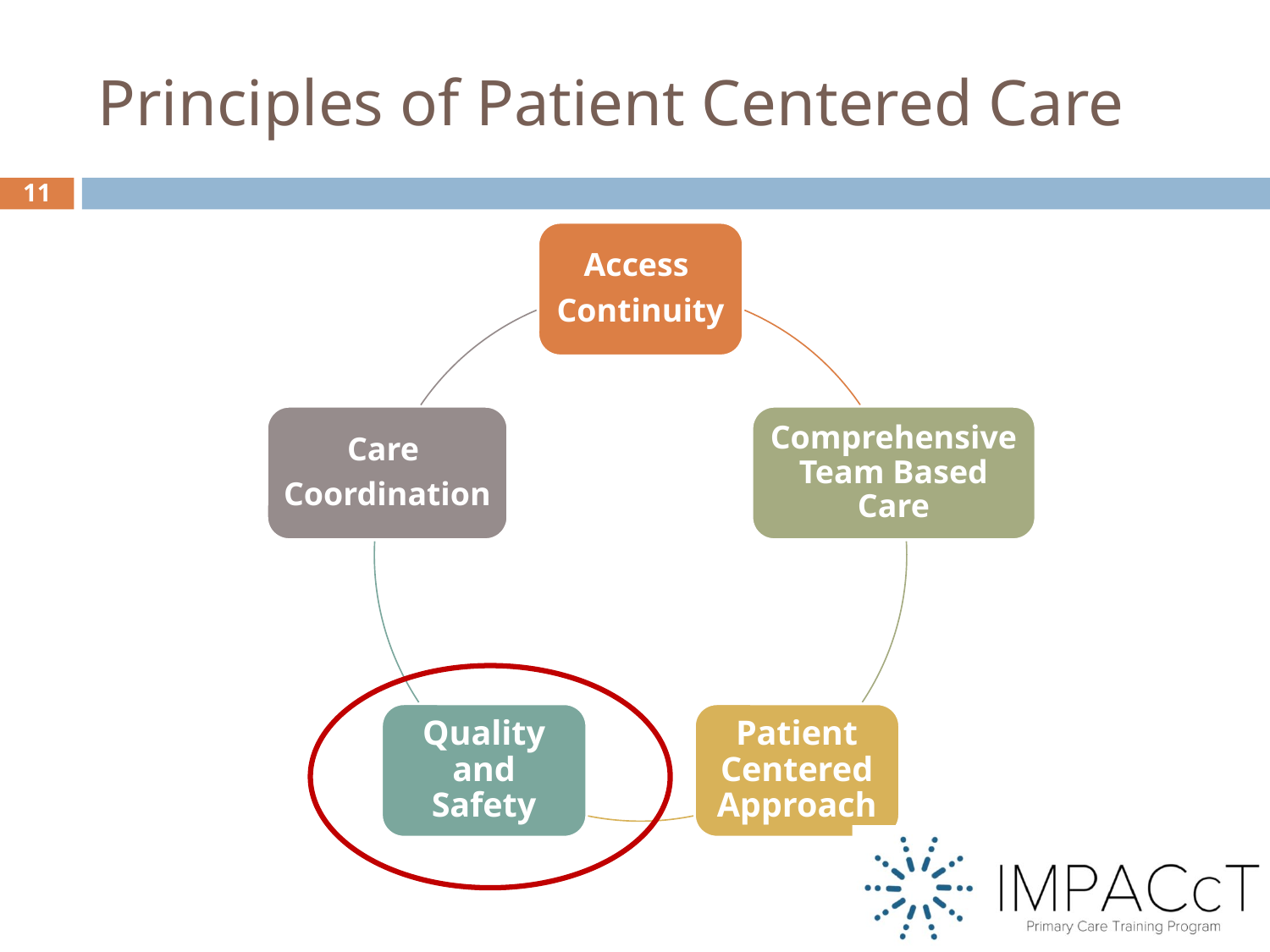

# Principles of Patient Centered Care
11
Access
Continuity
Care
Coordination
Comprehensive Team Based Care
Quality and Safety
Patient Centered Approach

## Slide 12
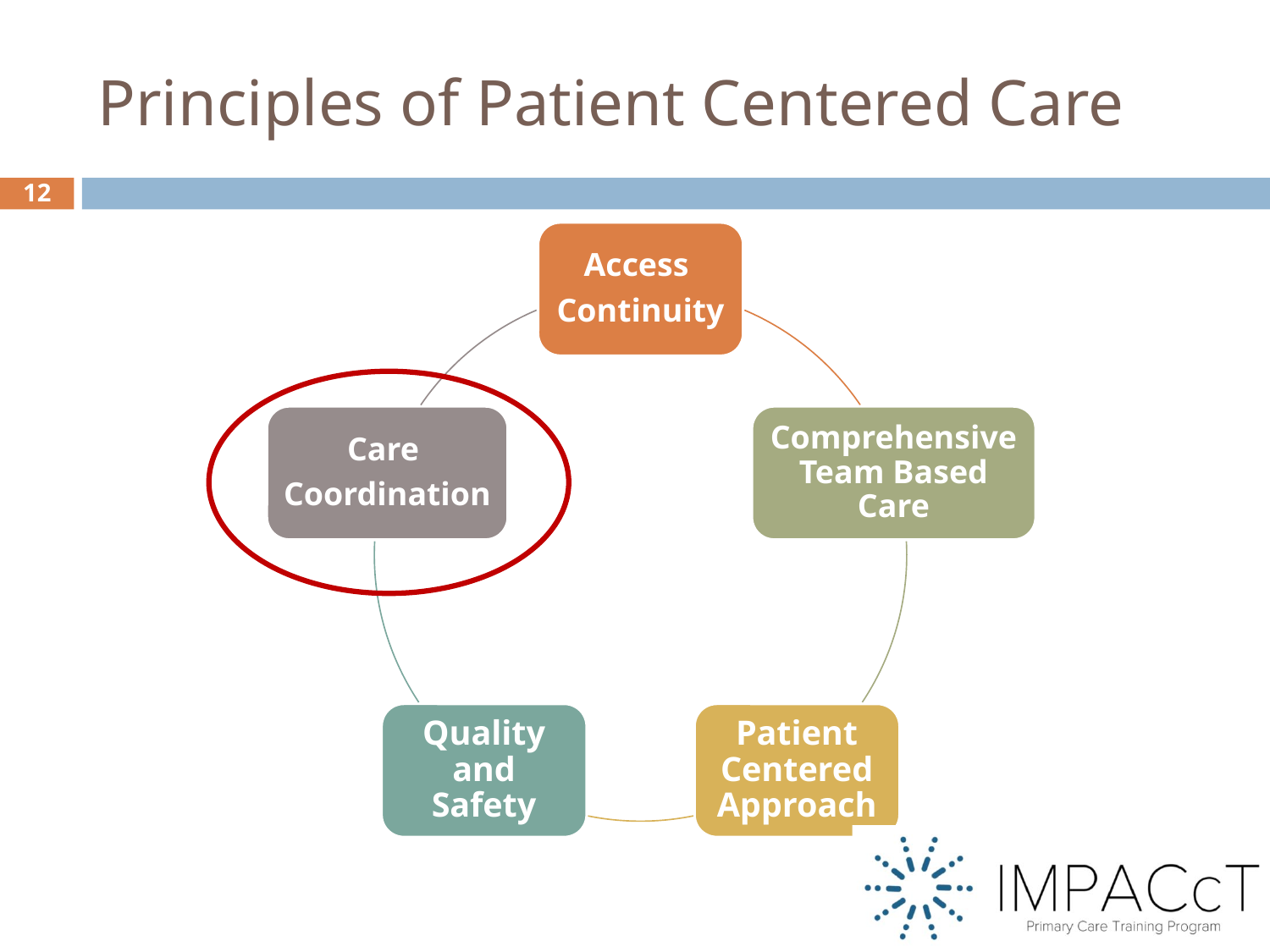

# Principles of Patient Centered Care
12
Access
Continuity
Care
Coordination
Comprehensive Team Based Care
Quality and Safety
Patient Centered Approach

## Slide 13
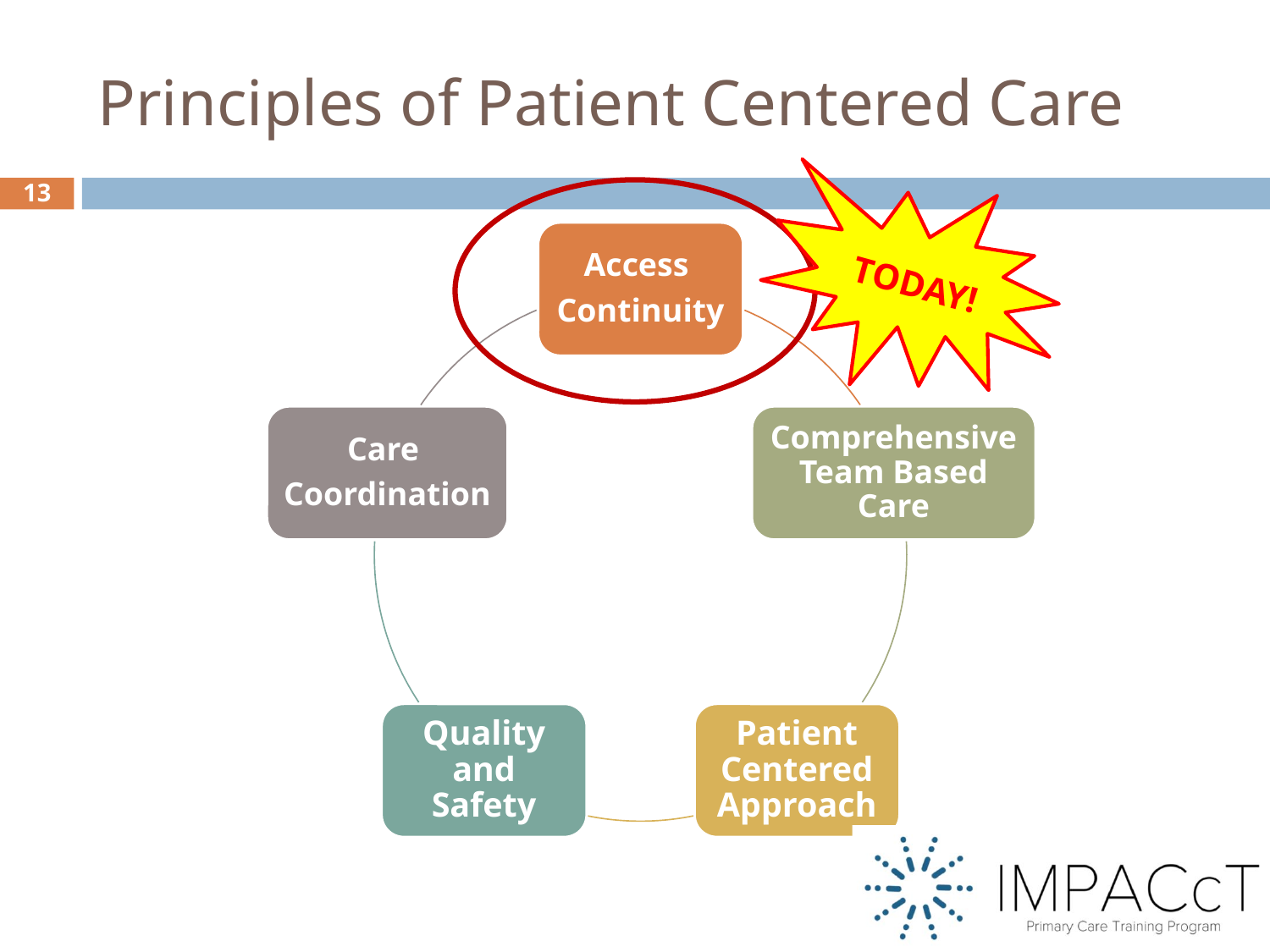

# Principles of Patient Centered Care
13
Access
Continuity
Care
Coordination
Comprehensive Team Based Care
Quality and Safety
Patient Centered Approach
TODAY!

## Slide 14
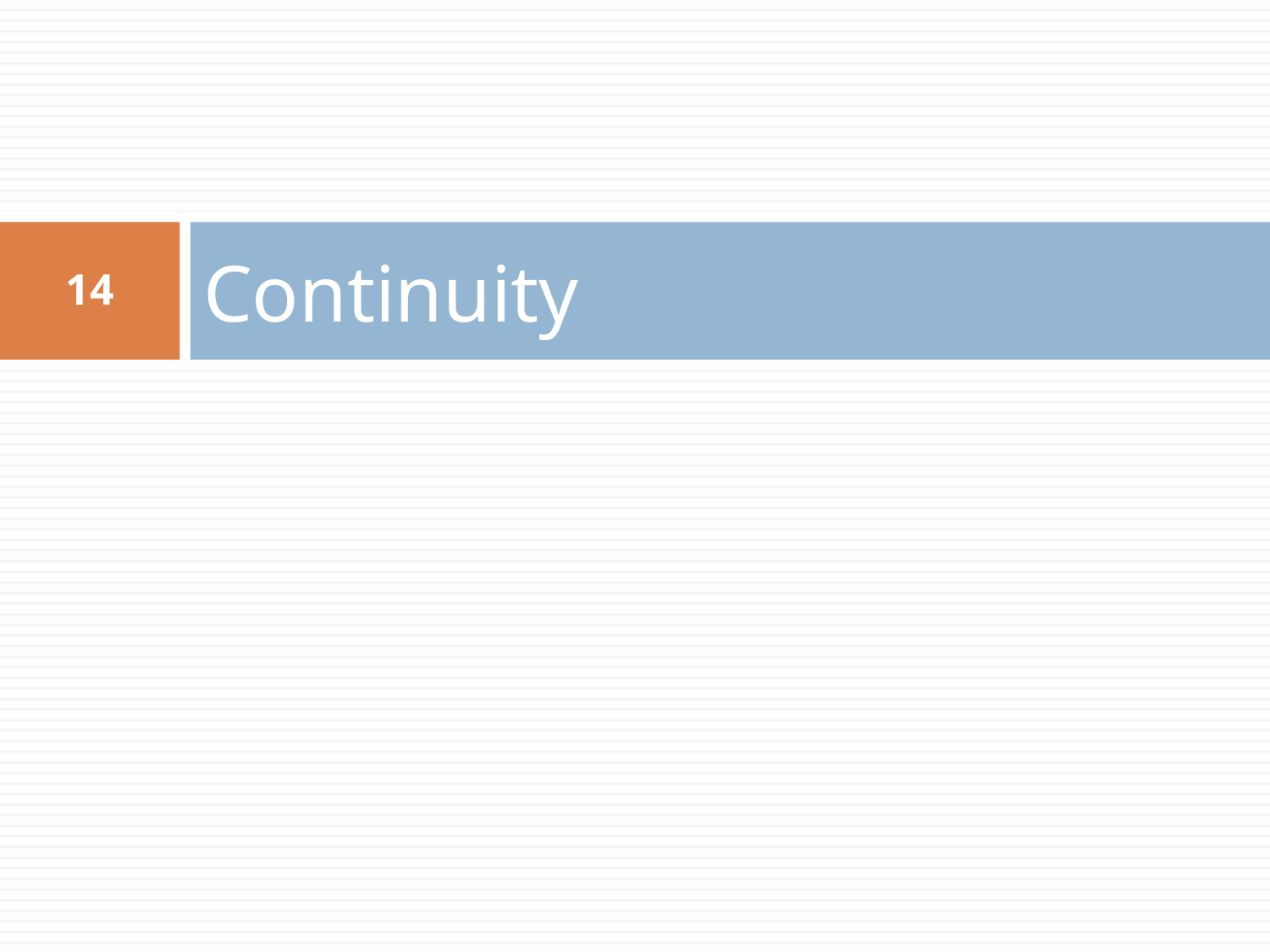

# Continuity
14

## Slide 15
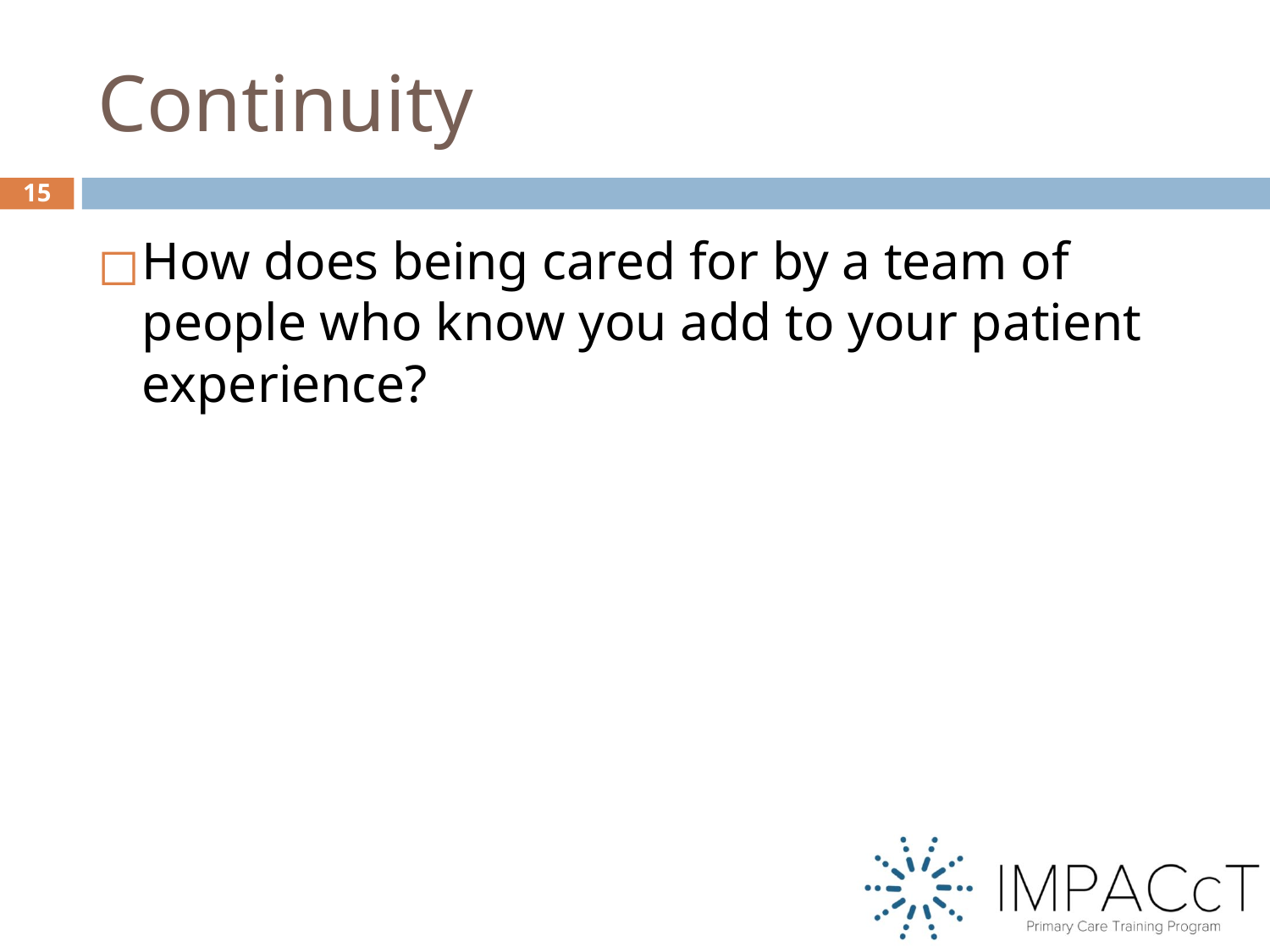

# Continuity
15
How does being cared for by a team of people who know you add to your patient experience?

## Slide 16
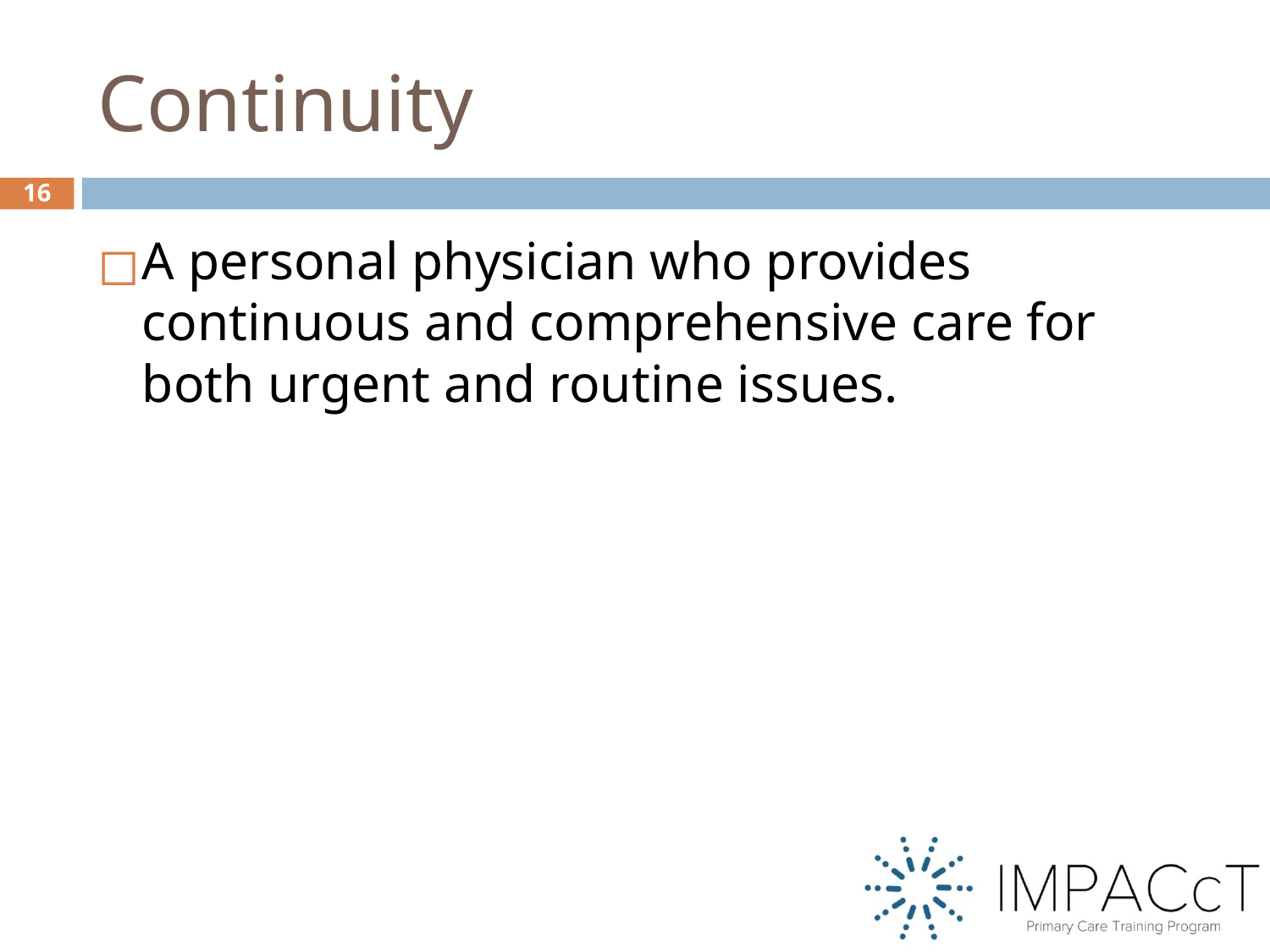

# Continuity
16
A personal physician who provides continuous and comprehensive care for both urgent and routine issues.

## Slide 17
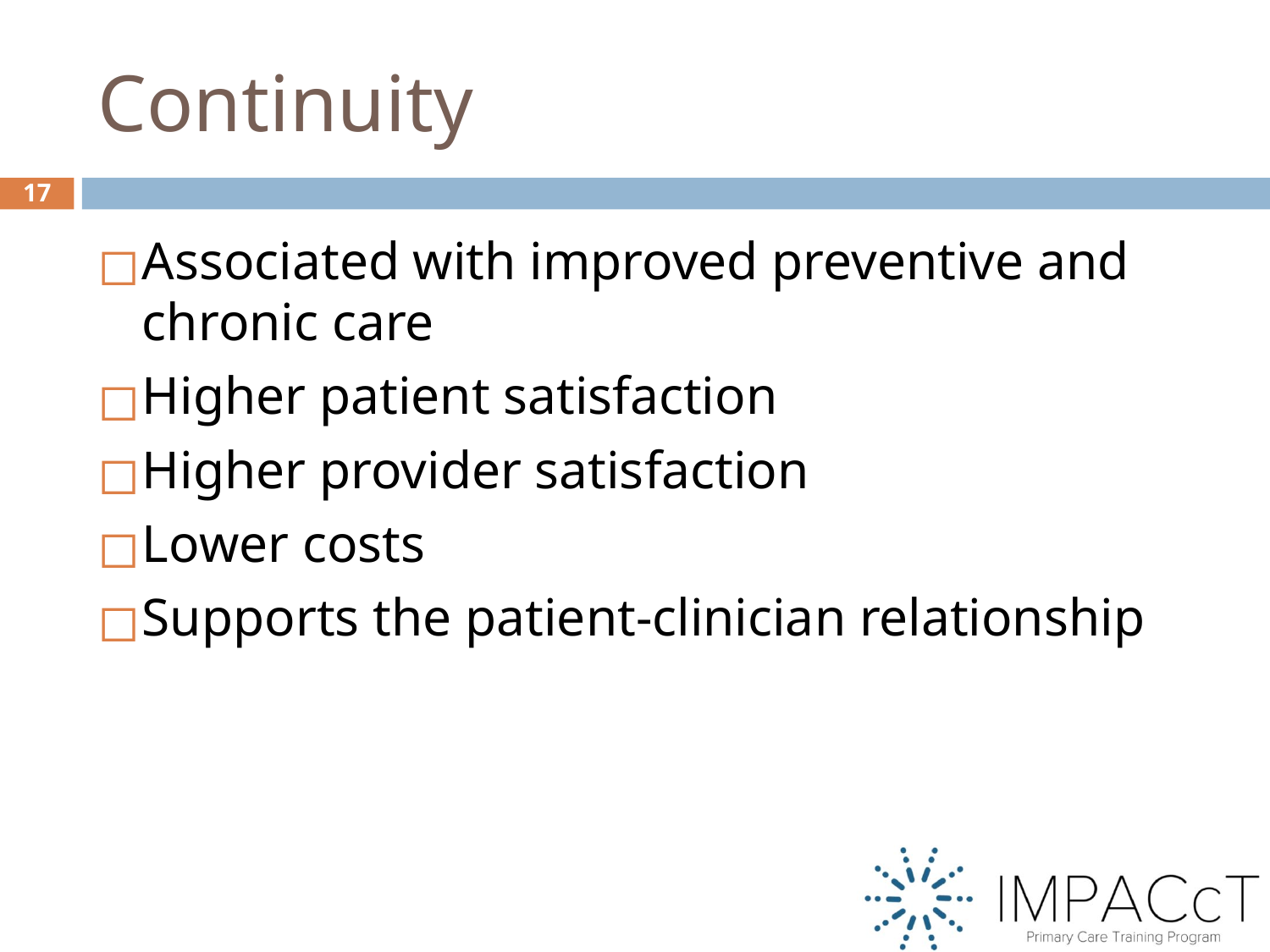

# Continuity
17
Associated with improved preventive and chronic care
Higher patient satisfaction
Higher provider satisfaction
Lower costs
Supports the patient-clinician relationship

## Slide 18
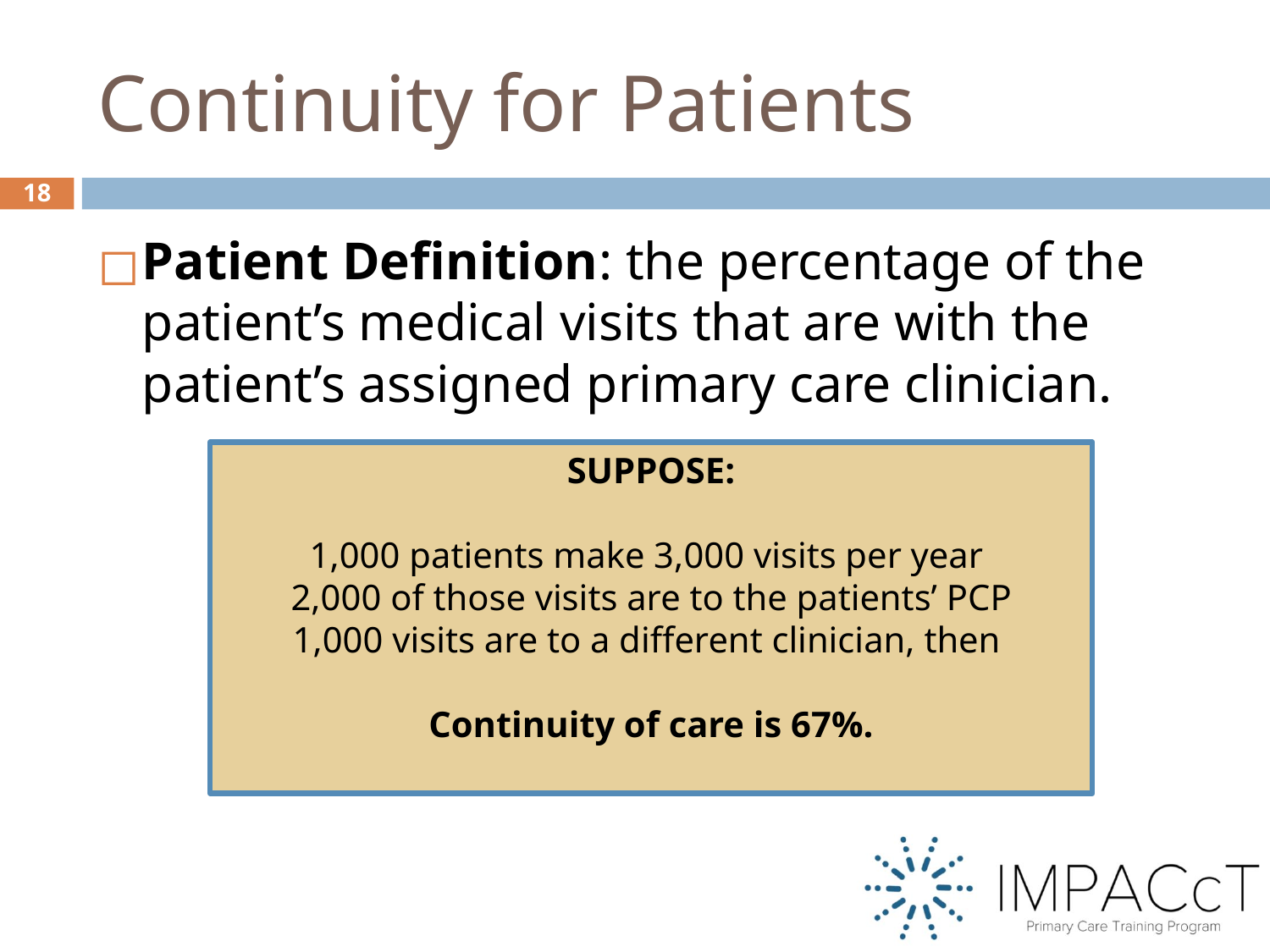

# Continuity for Patients
18
Patient Definition: the percentage of the patient’s medical visits that are with the patient’s assigned primary care clinician.
SUPPOSE:
1,000 patients make 3,000 visits per year
2,000 of those visits are to the patients’ PCP
1,000 visits are to a different clinician, then
Continuity of care is 67%.

## Slide 19
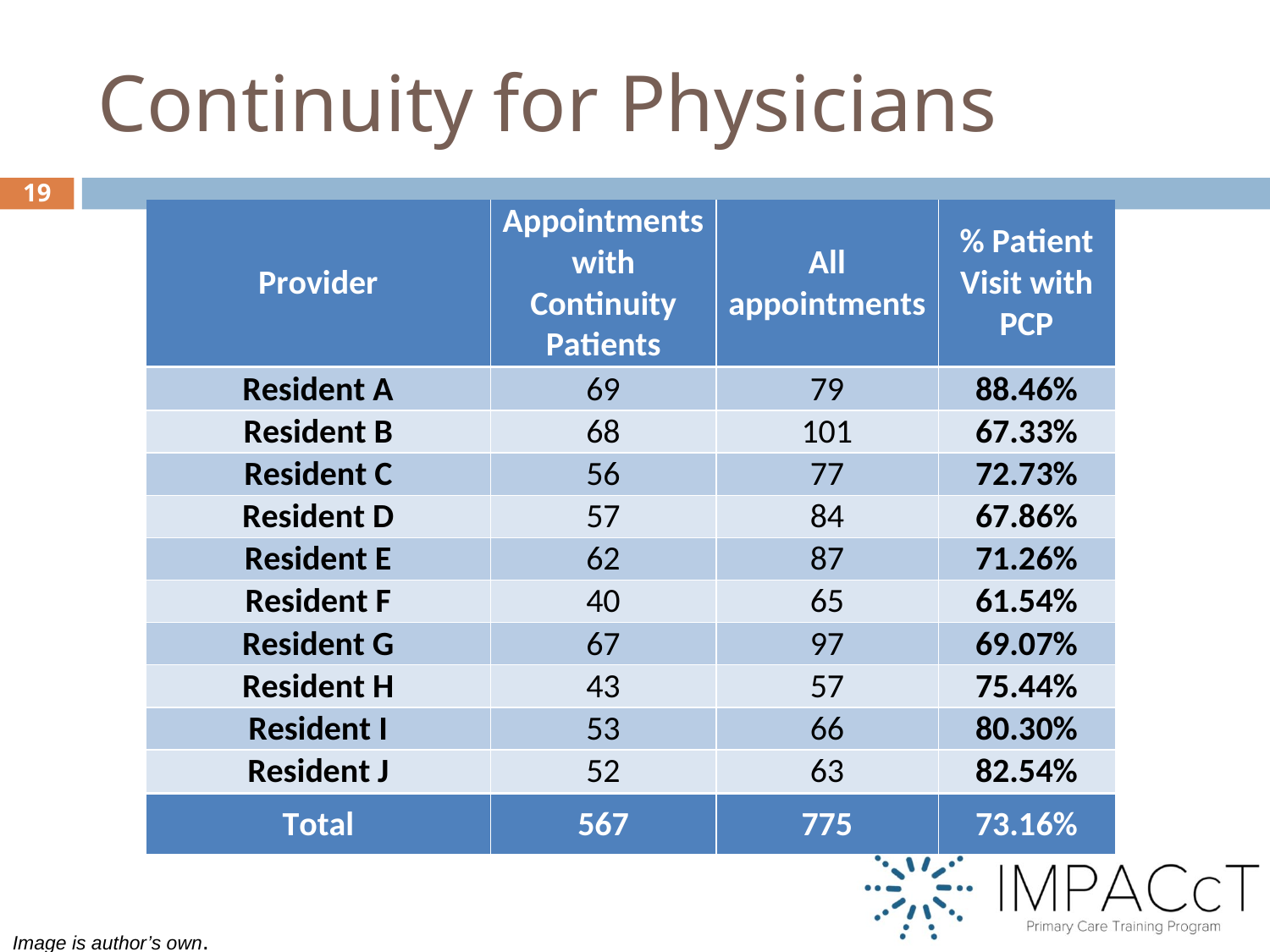

# Continuity for Physicians
19
Image is author’s own.

## Slide 20
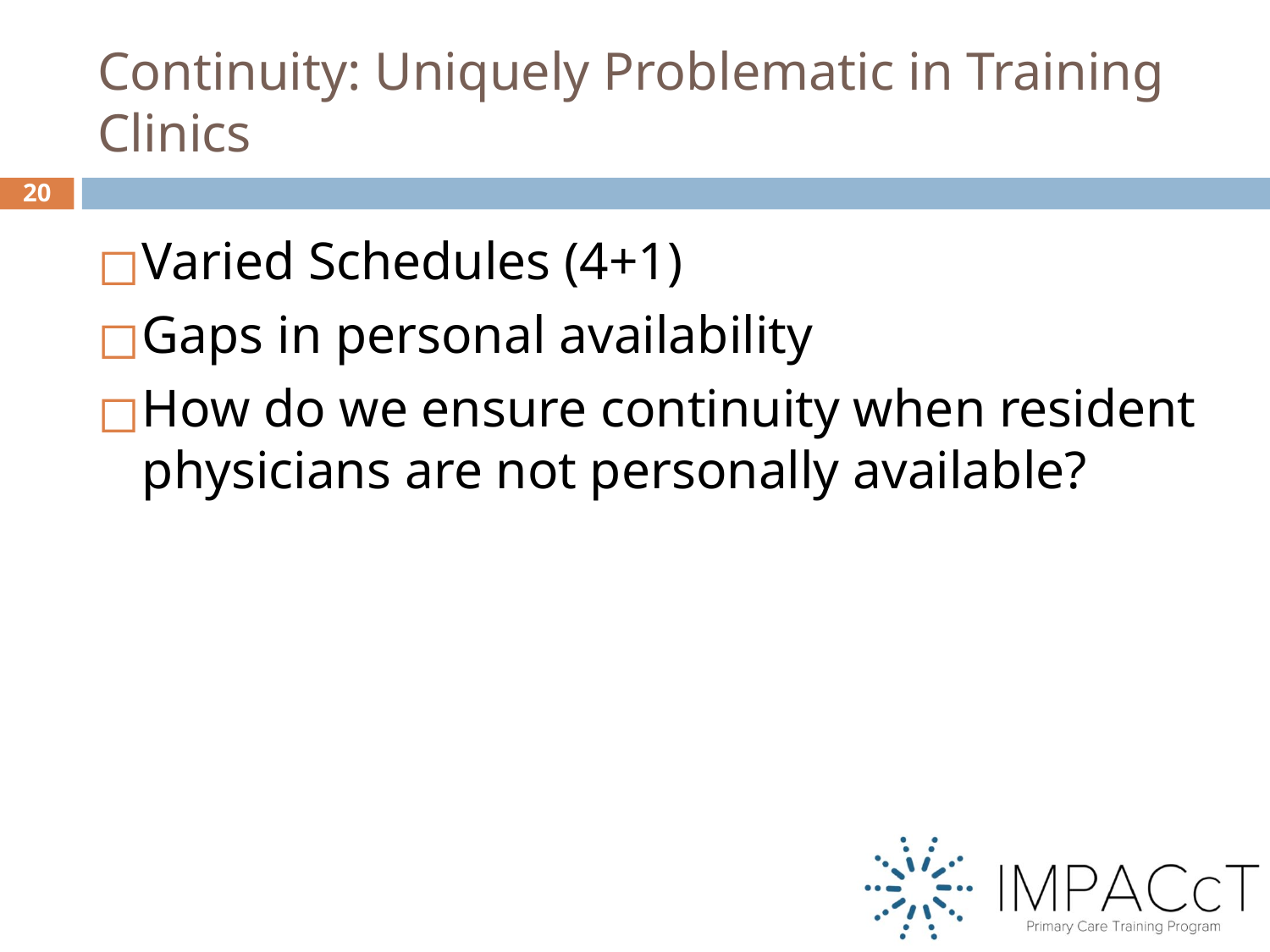

# Continuity: Uniquely Problematic in Training Clinics
20
Varied Schedules (4+1)
Gaps in personal availability
How do we ensure continuity when resident physicians are not personally available?

## Slide 21
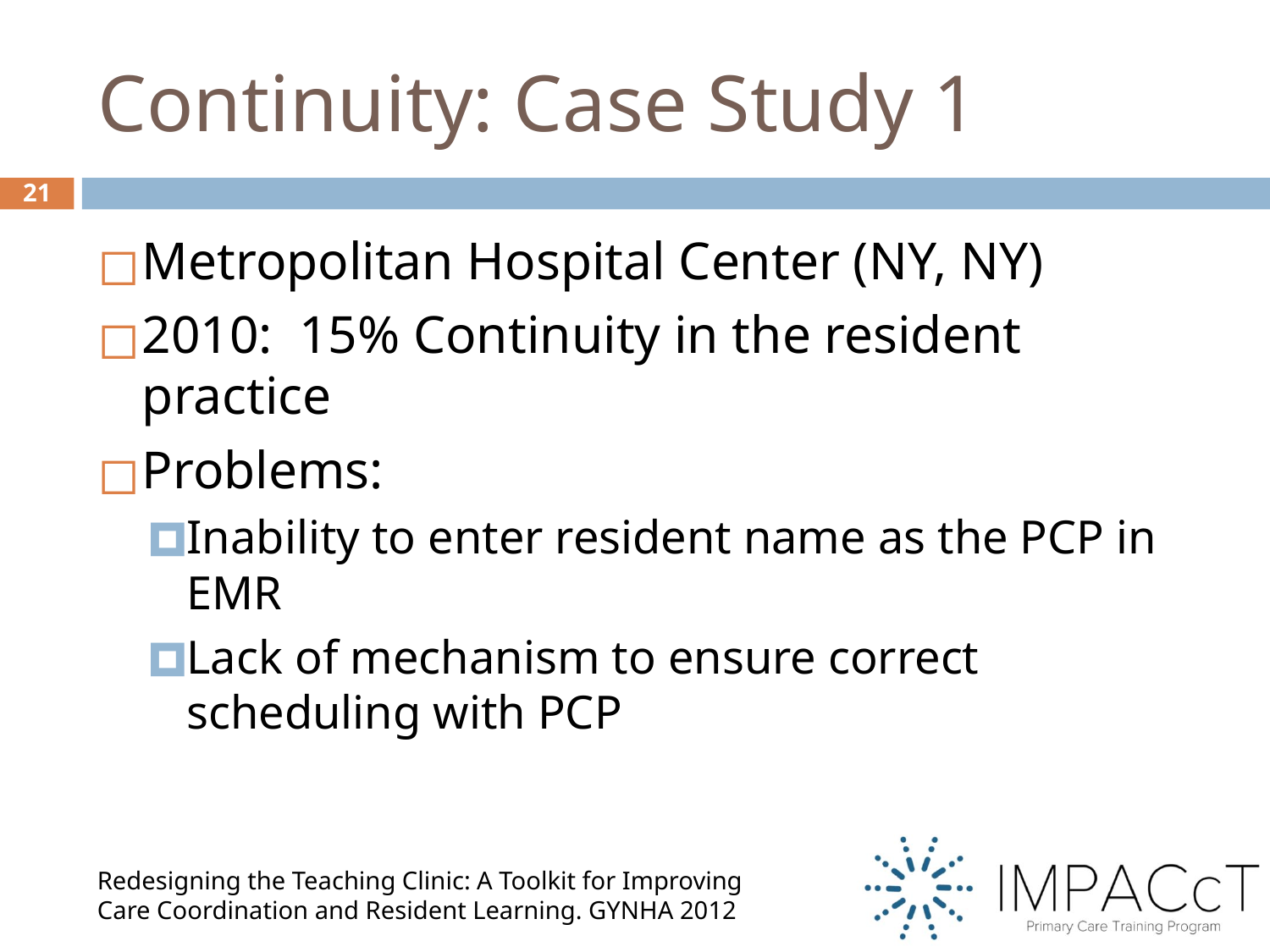

# Continuity: Case Study 1
21
Metropolitan Hospital Center (NY, NY)
2010: 15% Continuity in the resident practice
Problems:
Inability to enter resident name as the PCP in EMR
Lack of mechanism to ensure correct scheduling with PCP
Redesigning the Teaching Clinic: A Toolkit for Improving Care Coordination and Resident Learning. GYNHA 2012

## Slide 22
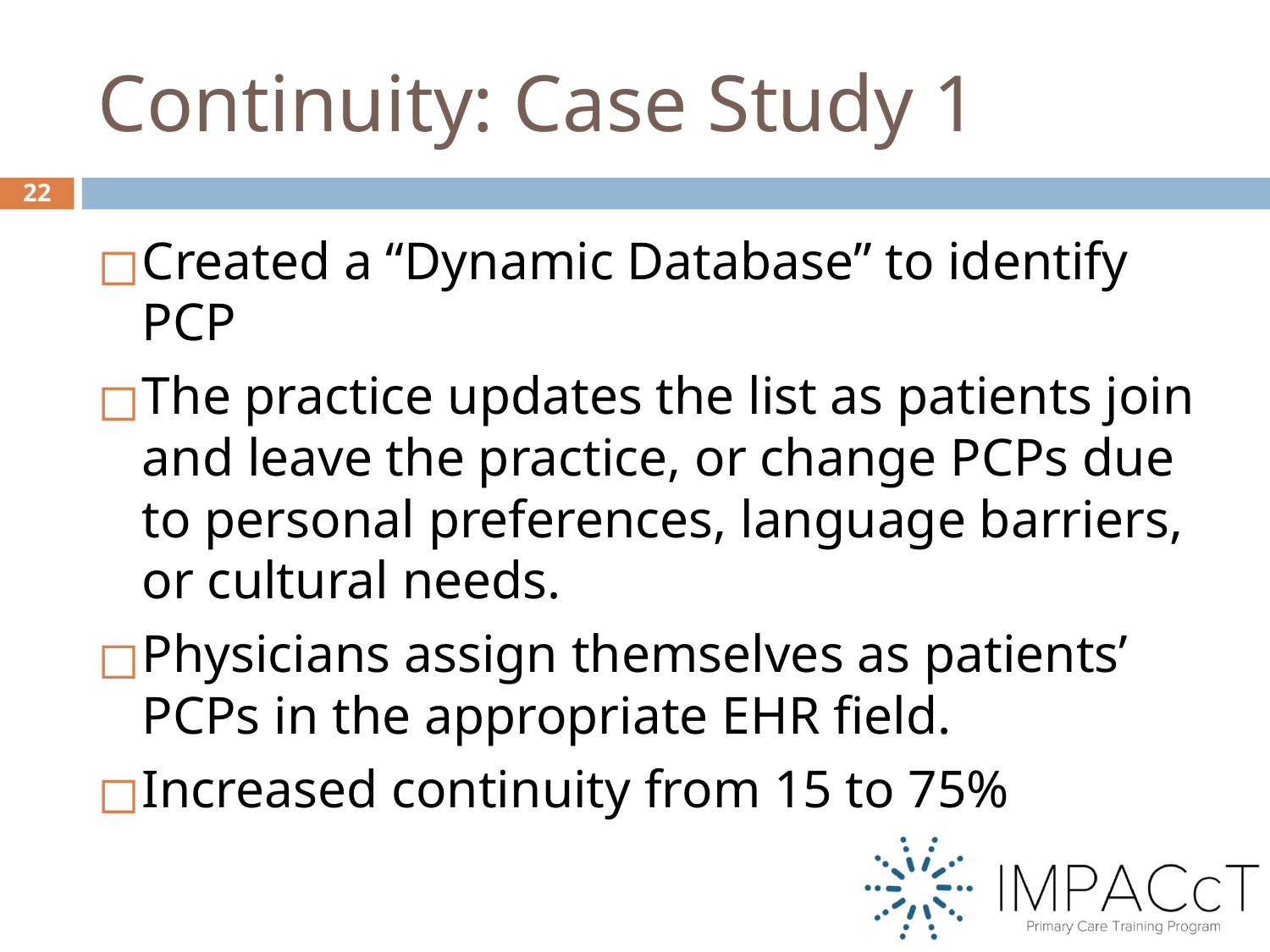

# Continuity: Case Study 1
22
Created a “Dynamic Database” to identify PCP
The practice updates the list as patients join and leave the practice, or change PCPs due to personal preferences, language barriers, or cultural needs.
Physicians assign themselves as patients’ PCPs in the appropriate EHR field.
Increased continuity from 15 to 75%

## Slide 23
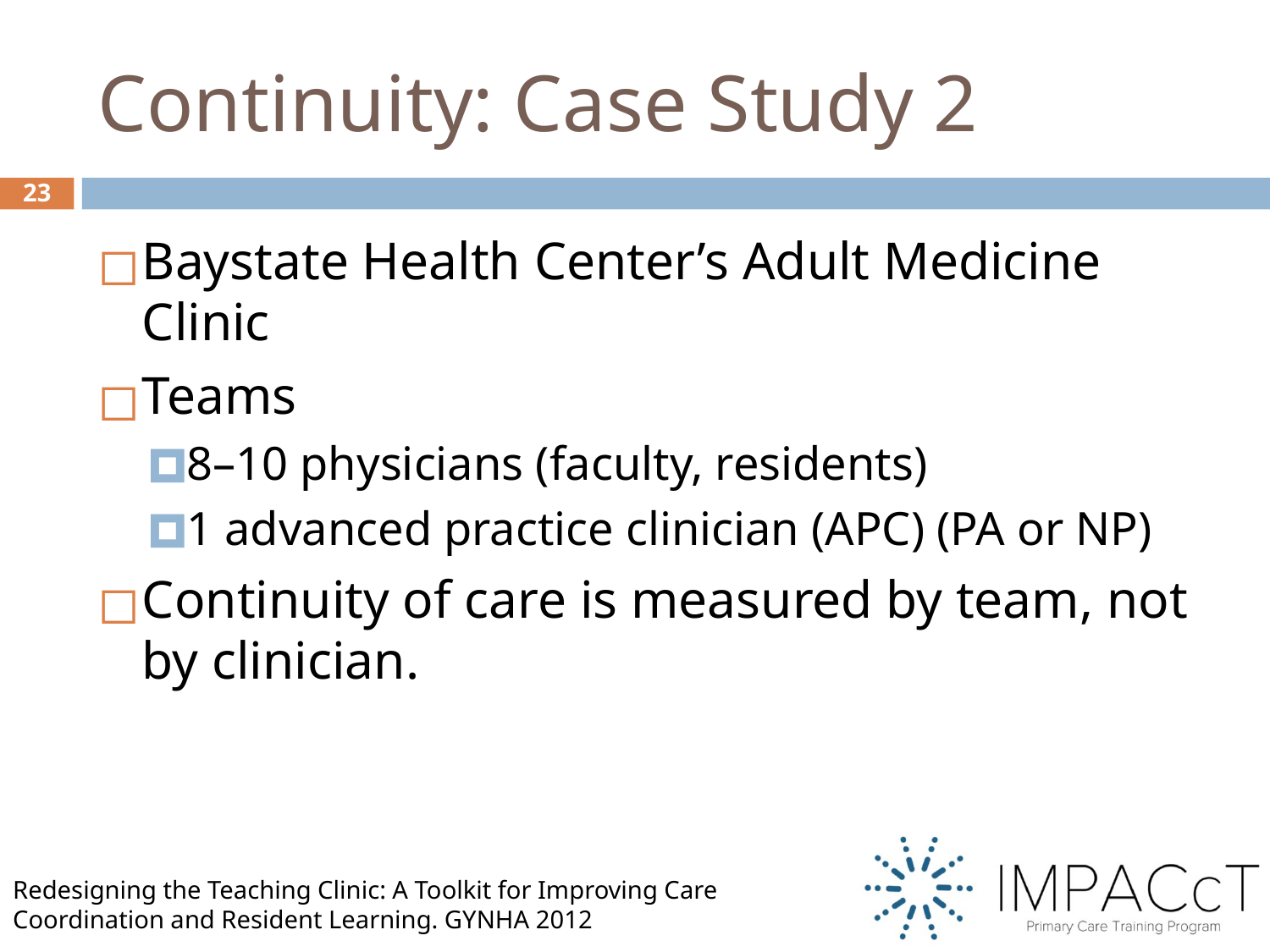

# Continuity: Case Study 2
23
Baystate Health Center’s Adult Medicine Clinic
Teams
8–10 physicians (faculty, residents)
1 advanced practice clinician (APC) (PA or NP)
Continuity of care is measured by team, not by clinician.
Redesigning the Teaching Clinic: A Toolkit for Improving Care Coordination and Resident Learning. GYNHA 2012

## Slide 24
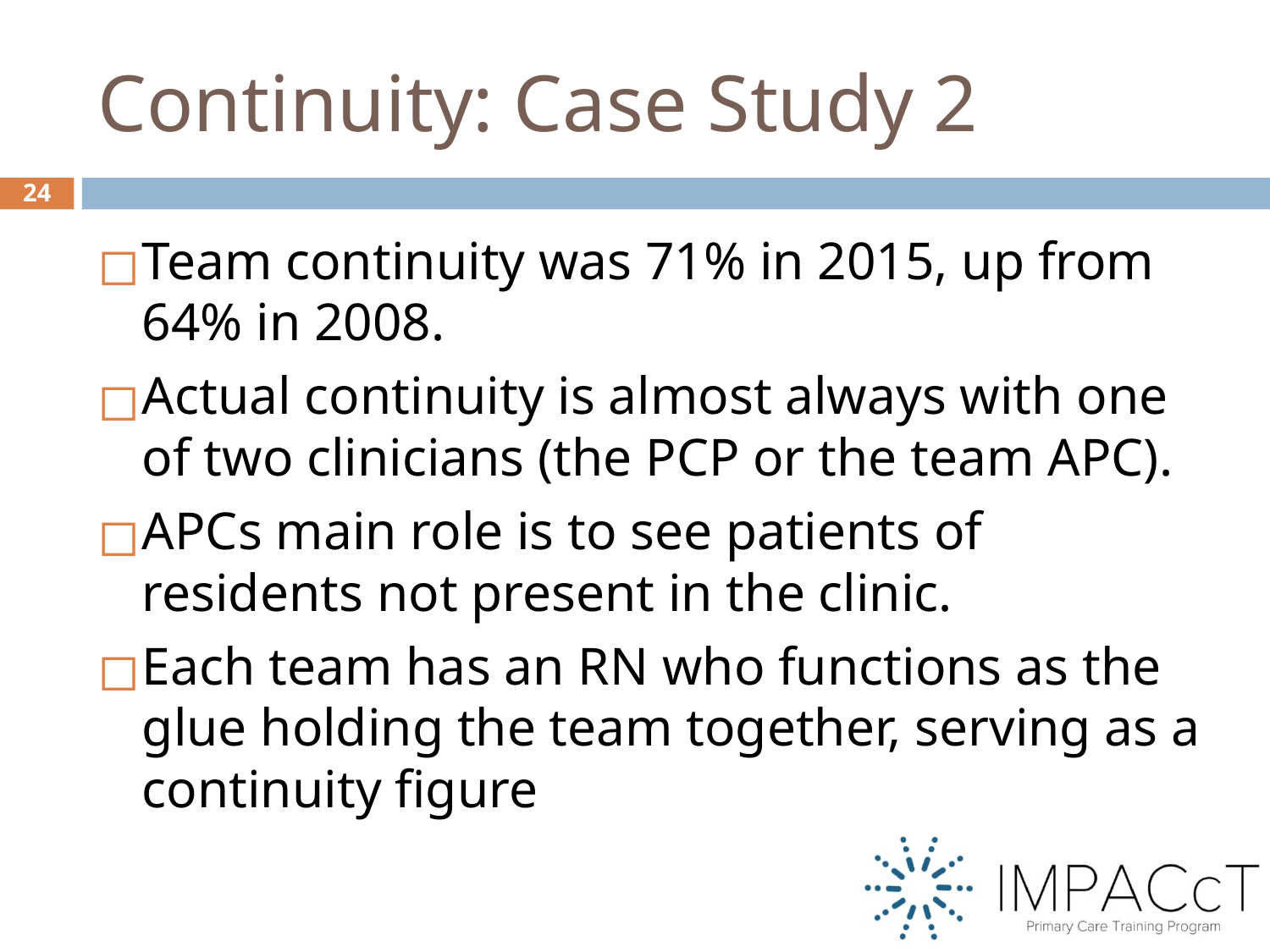

# Continuity: Case Study 2
24
Team continuity was 71% in 2015, up from 64% in 2008.
Actual continuity is almost always with one of two clinicians (the PCP or the team APC).
APCs main role is to see patients of residents not present in the clinic.
Each team has an RN who functions as the glue holding the team together, serving as a continuity figure

## Slide 25
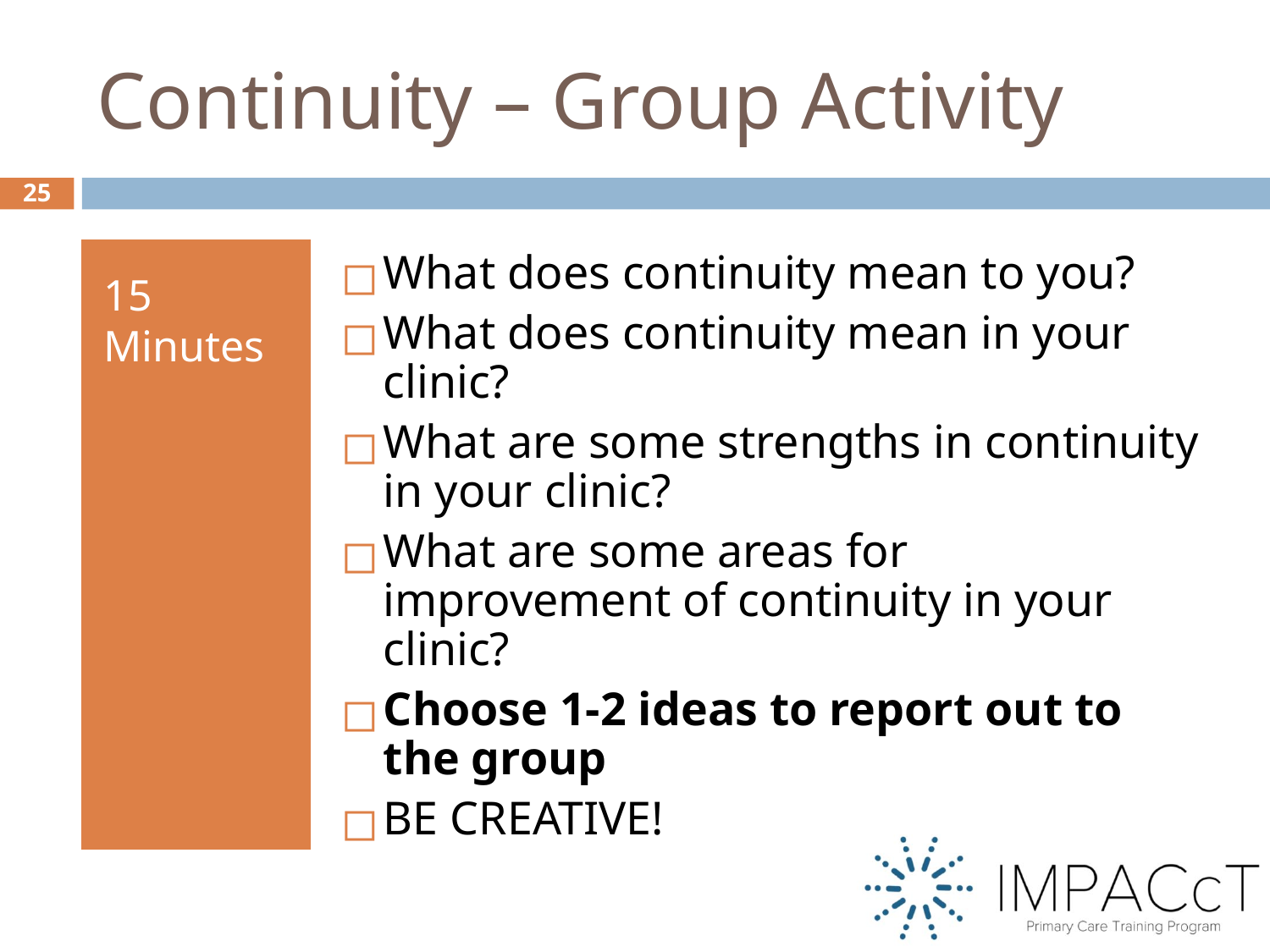

# Continuity – Group Activity
25
15 Minutes
What does continuity mean to you?
What does continuity mean in your clinic?
What are some strengths in continuity in your clinic?
What are some areas for improvement of continuity in your clinic?
Choose 1-2 ideas to report out to the group
BE CREATIVE!

## Slide 26
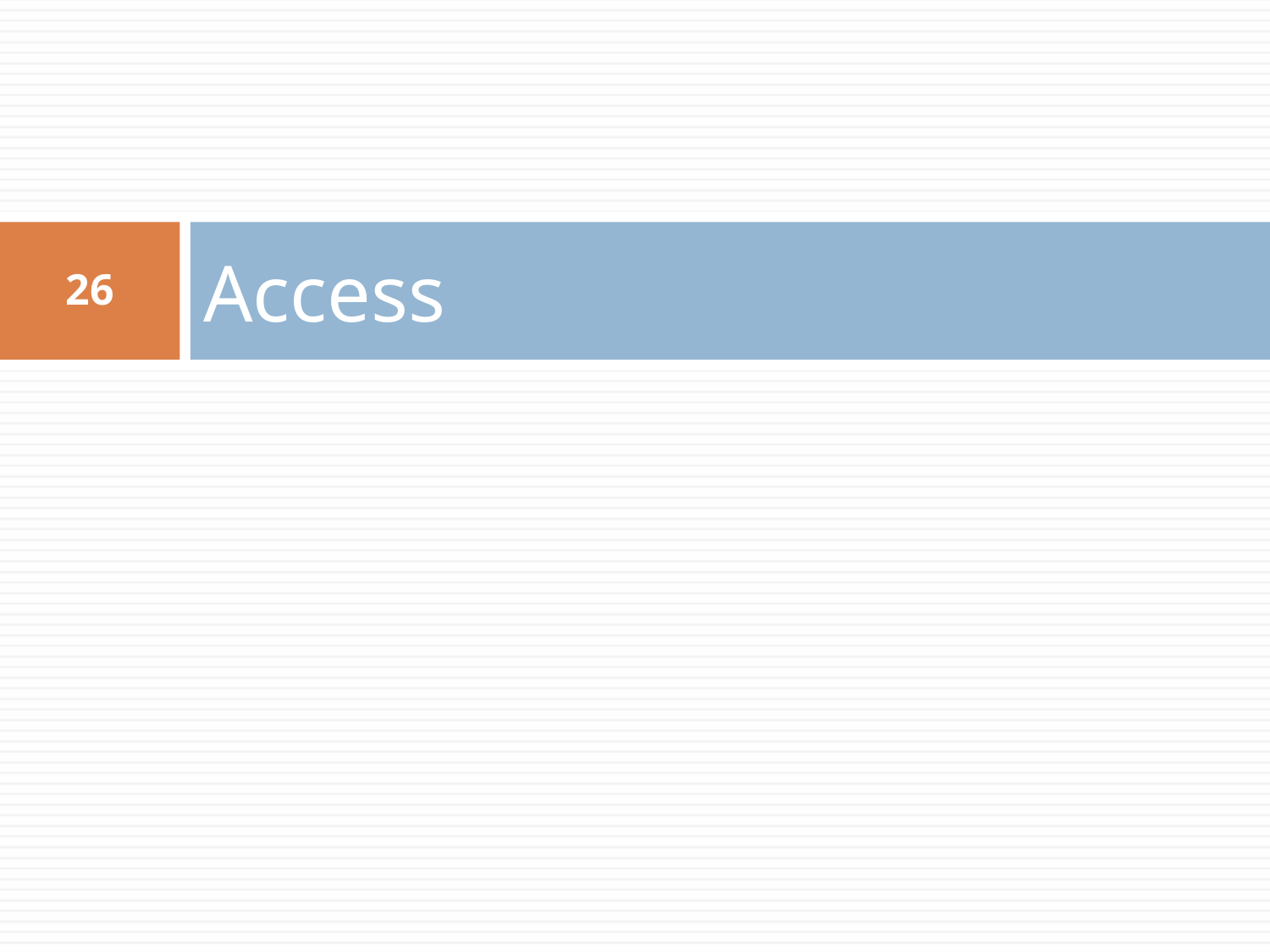

# Access
26

## Slide 27
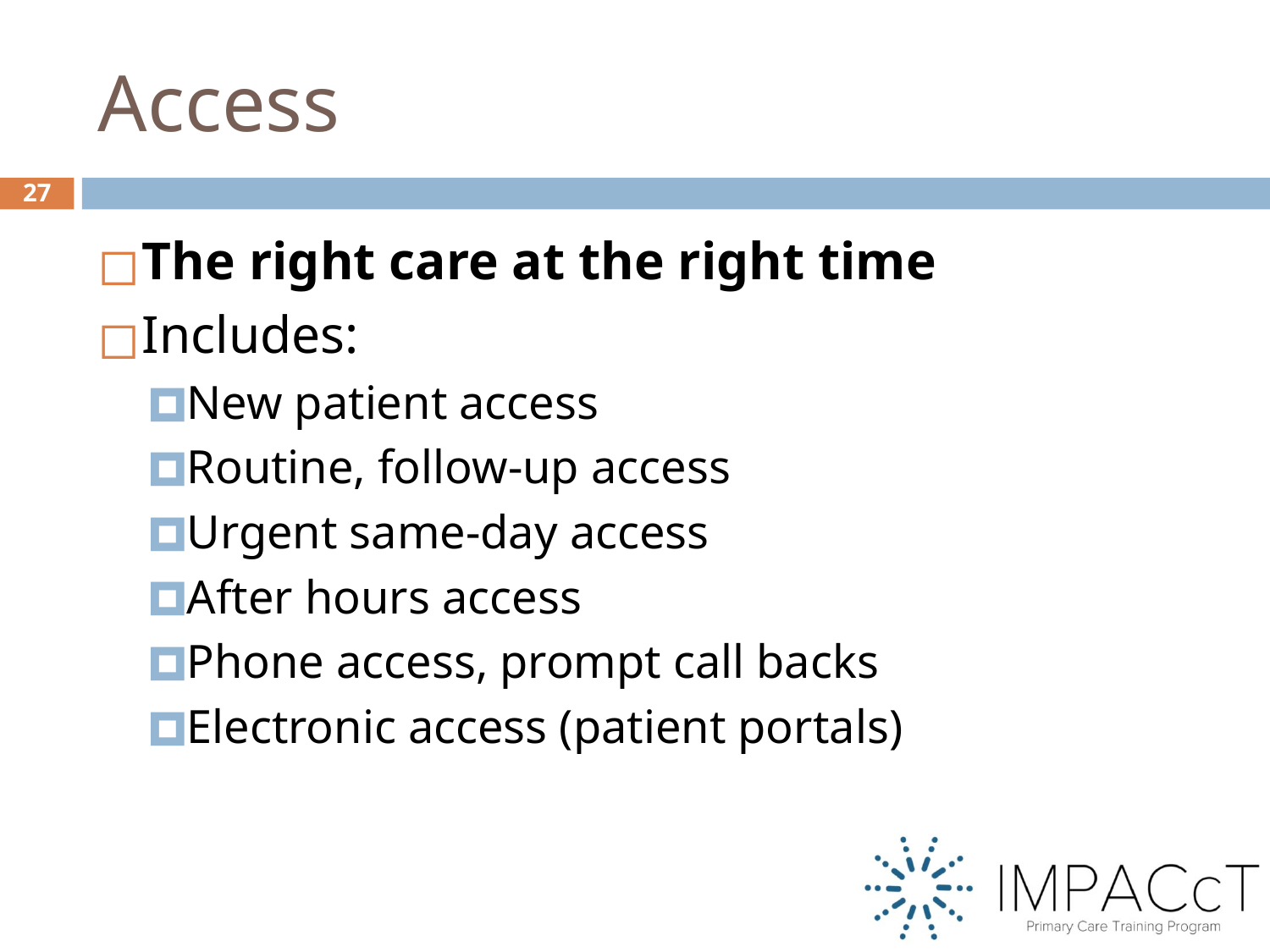

# Access
27
The right care at the right time
Includes:
New patient access
Routine, follow-up access
Urgent same-day access
After hours access
Phone access, prompt call backs
Electronic access (patient portals)

## Slide 28
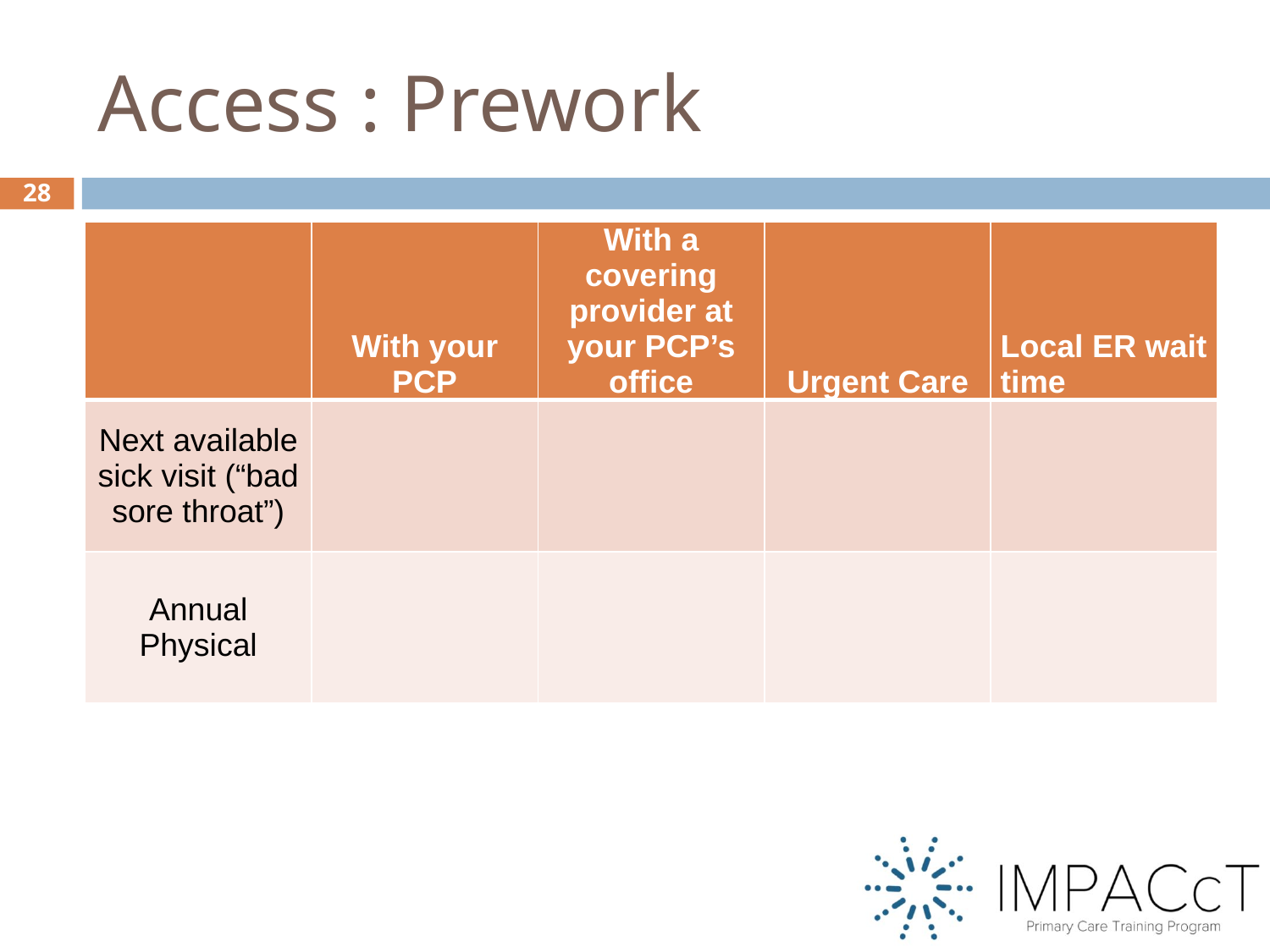

# Access : Prework
28
| | With your PCP | With a covering provider at your PCP’s office | Urgent Care | Local ER wait time |
| --- | --- | --- | --- | --- |
| Next available sick visit (“bad sore throat”) | | | | |
| Annual Physical | | | | |

## Slide 29
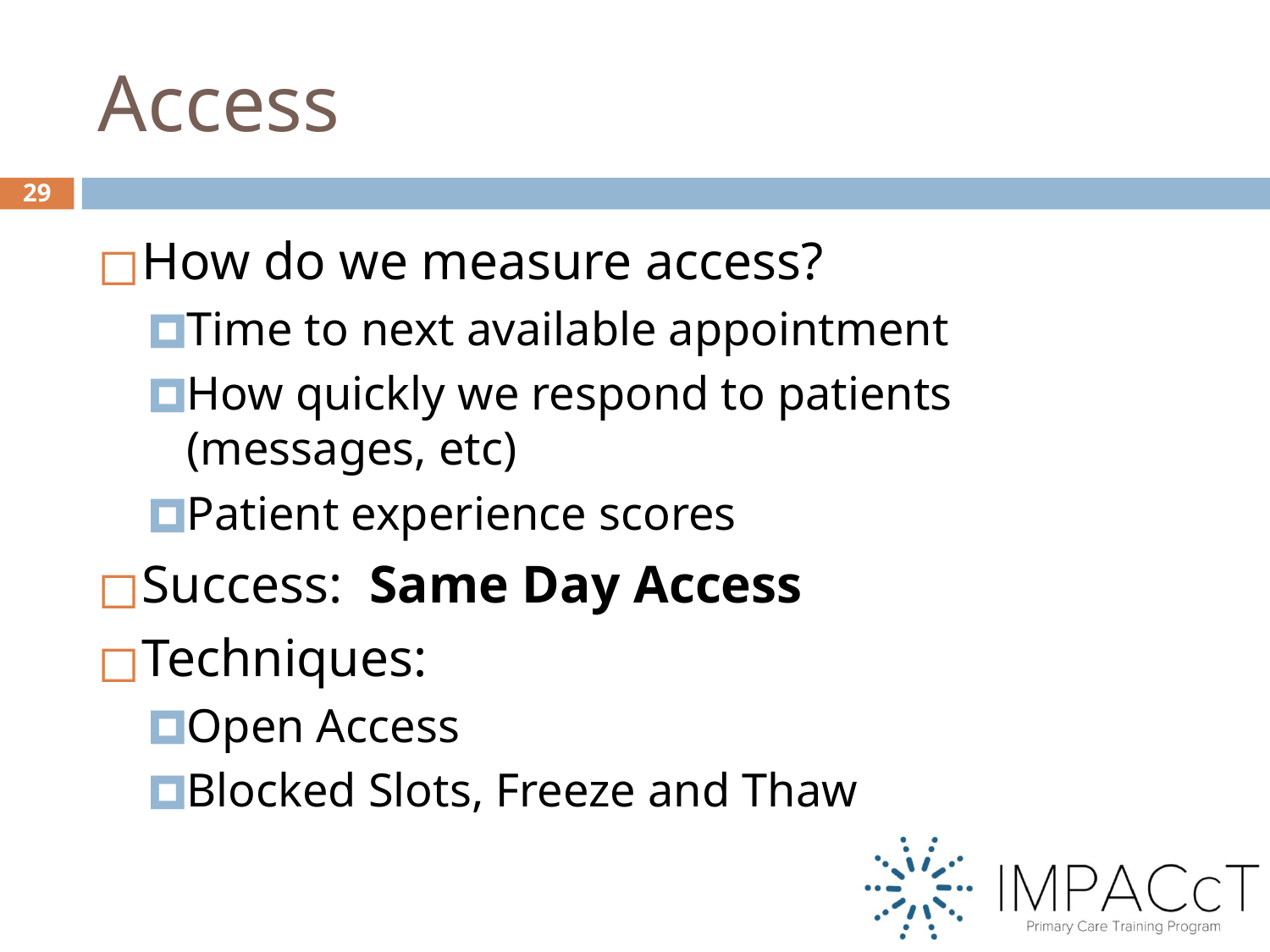

# Access
29
How do we measure access?
Time to next available appointment
How quickly we respond to patients (messages, etc)
Patient experience scores
Success: Same Day Access
Techniques:
Open Access
Blocked Slots, Freeze and Thaw

## Slide 30
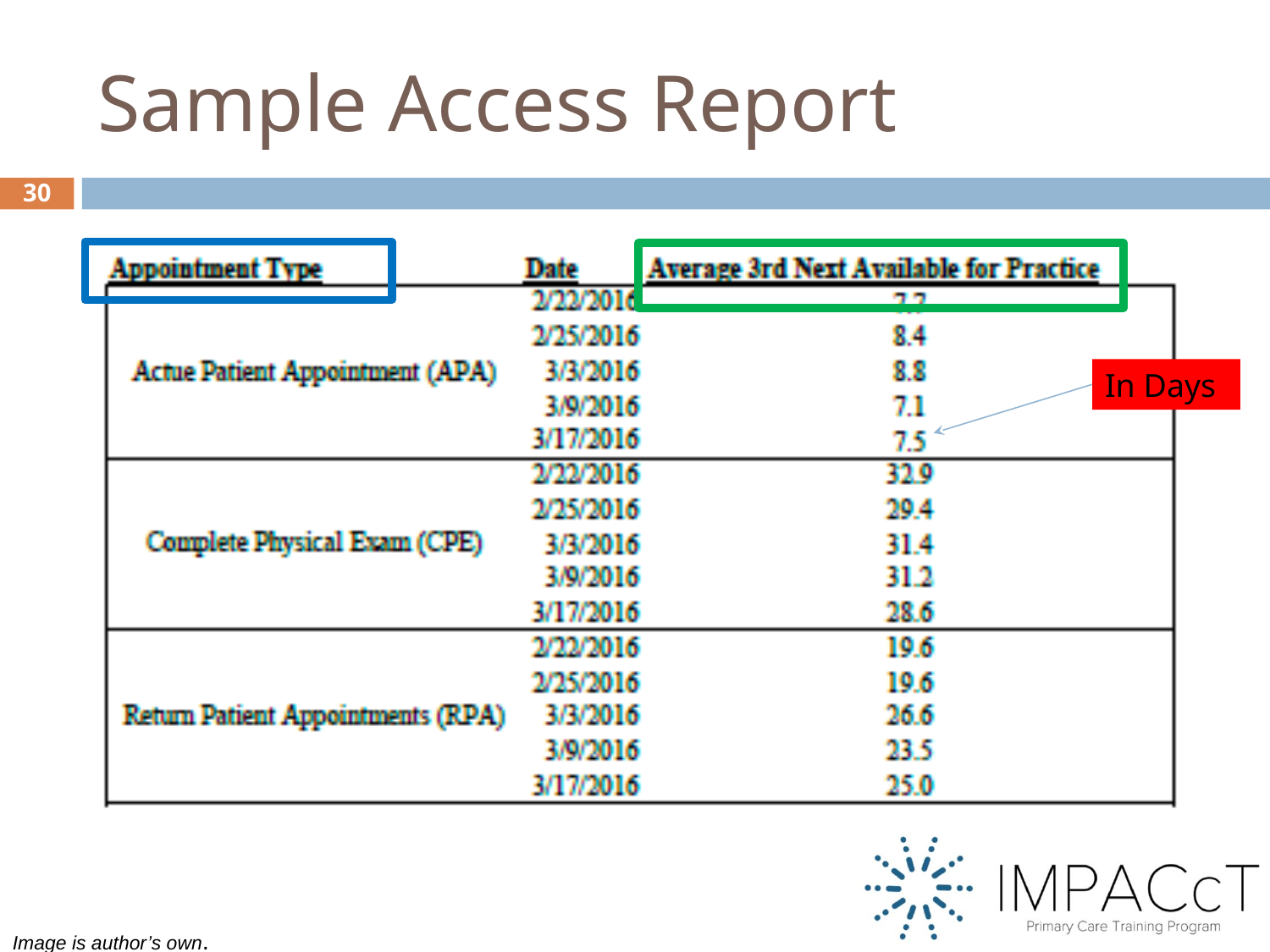

# Sample Access Report
30
In Days
Image is author’s own.

## Slide 31
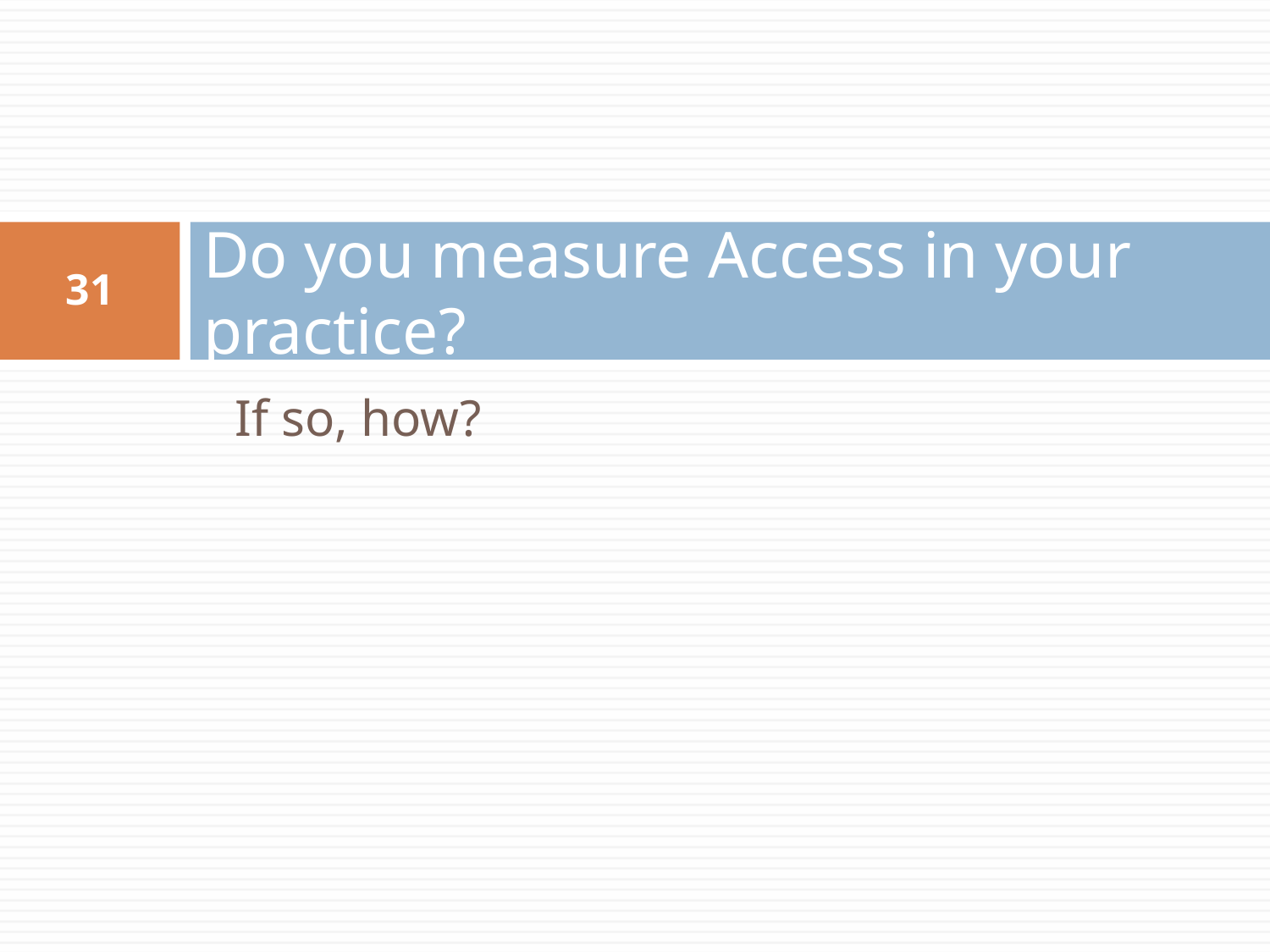

# Do you measure Access in your practice?
31
If so, how?

## Slide 32
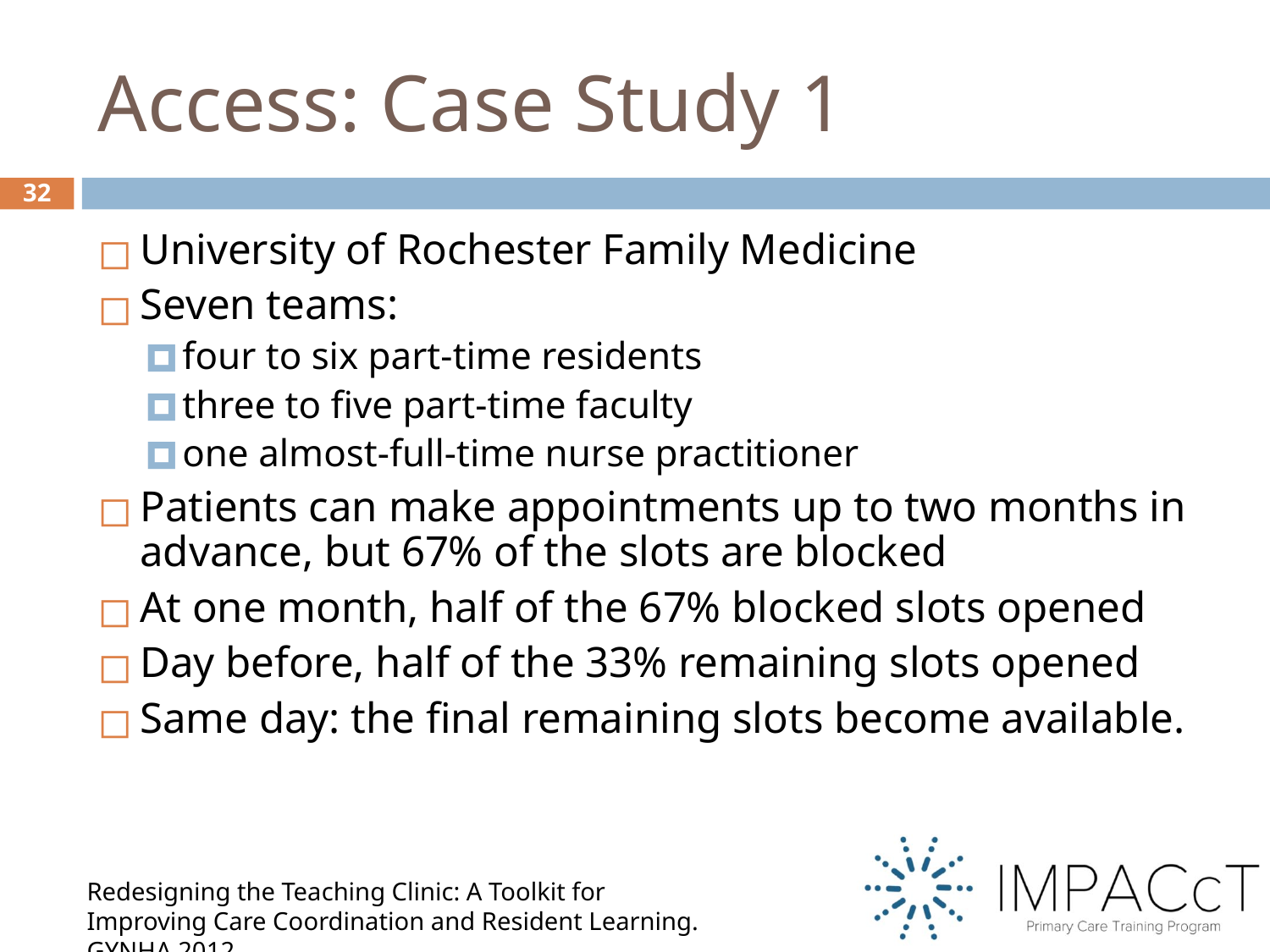

# Access: Case Study 1
32
University of Rochester Family Medicine
Seven teams:
four to six part-time residents
three to five part-time faculty
one almost-full-time nurse practitioner
Patients can make appointments up to two months in advance, but 67% of the slots are blocked
At one month, half of the 67% blocked slots opened
Day before, half of the 33% remaining slots opened
Same day: the final remaining slots become available.
Redesigning the Teaching Clinic: A Toolkit for Improving Care Coordination and Resident Learning. GYNHA 2012

## Slide 33
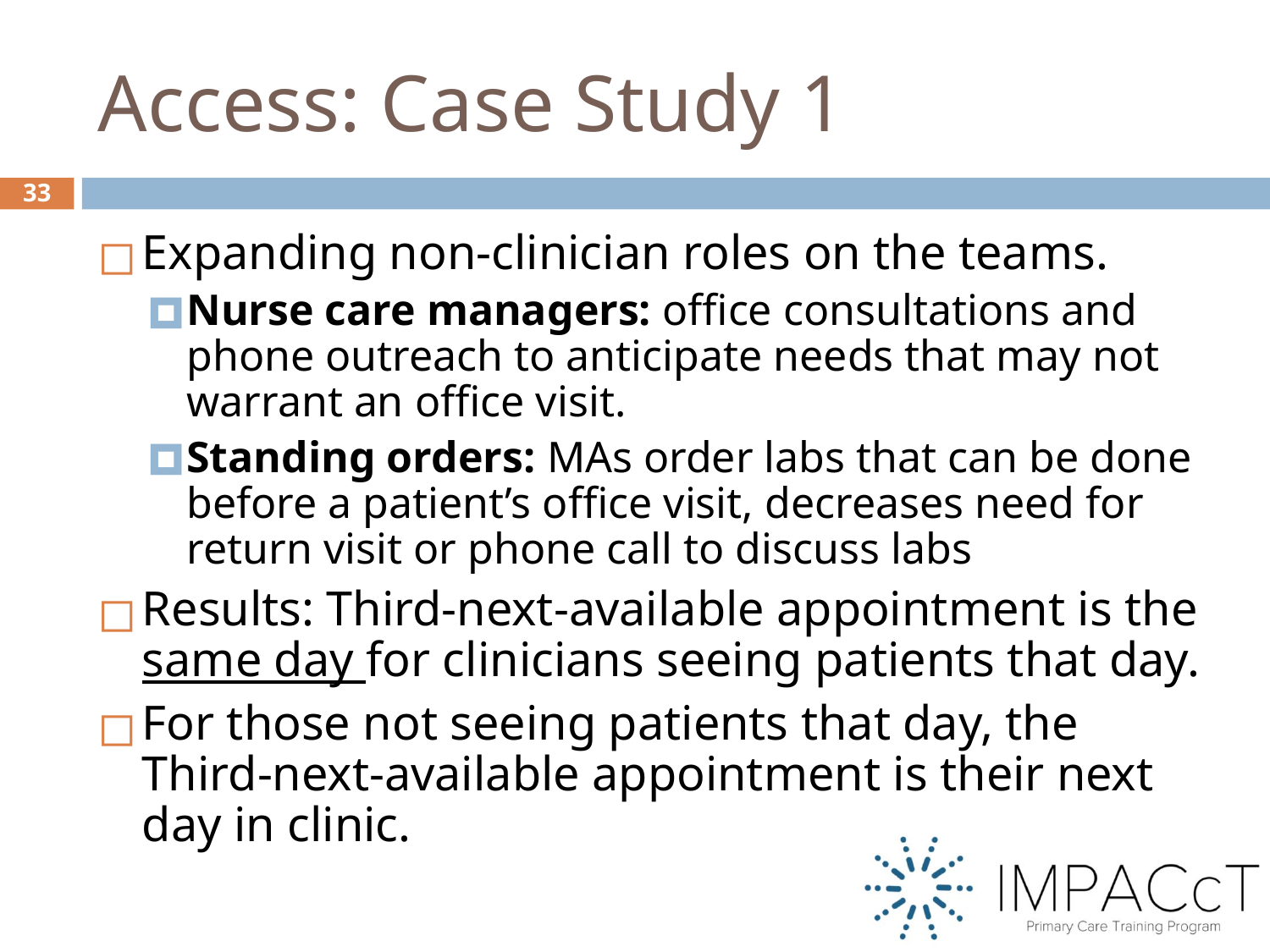

# Access: Case Study 1
33
Expanding non-clinician roles on the teams.
Nurse care managers: office consultations and phone outreach to anticipate needs that may not warrant an office visit.
Standing orders: MAs order labs that can be done before a patient’s office visit, decreases need for return visit or phone call to discuss labs
Results: Third-next-available appointment is the same day for clinicians seeing patients that day.
For those not seeing patients that day, the Third-next-available appointment is their next day in clinic.

## Slide 34
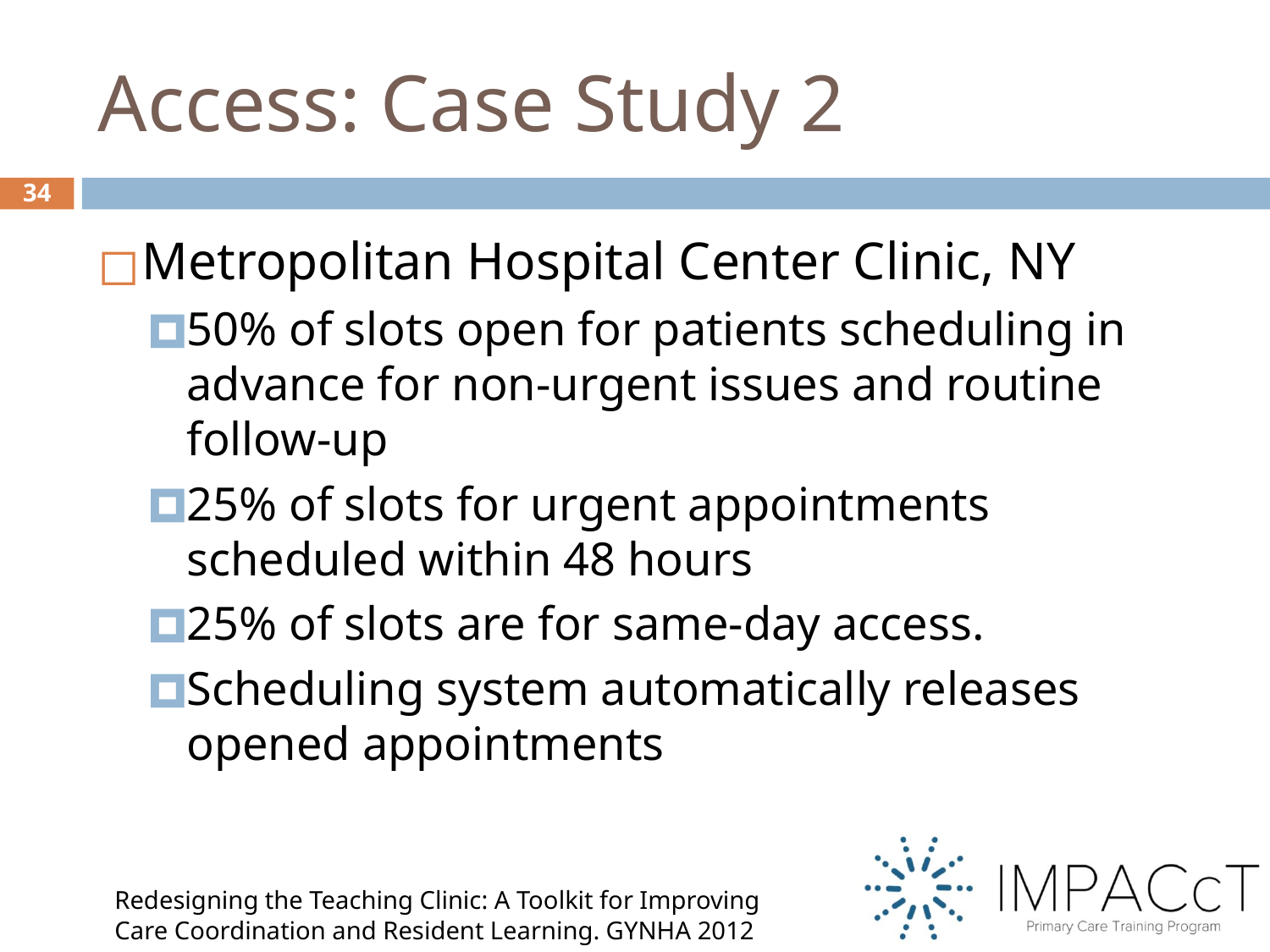

# Access: Case Study 2
34
Metropolitan Hospital Center Clinic, NY
50% of slots open for patients scheduling in advance for non-urgent issues and routine follow-up
25% of slots for urgent appointments scheduled within 48 hours
25% of slots are for same-day access.
Scheduling system automatically releases opened appointments
Redesigning the Teaching Clinic: A Toolkit for Improving Care Coordination and Resident Learning. GYNHA 2012

## Slide 35
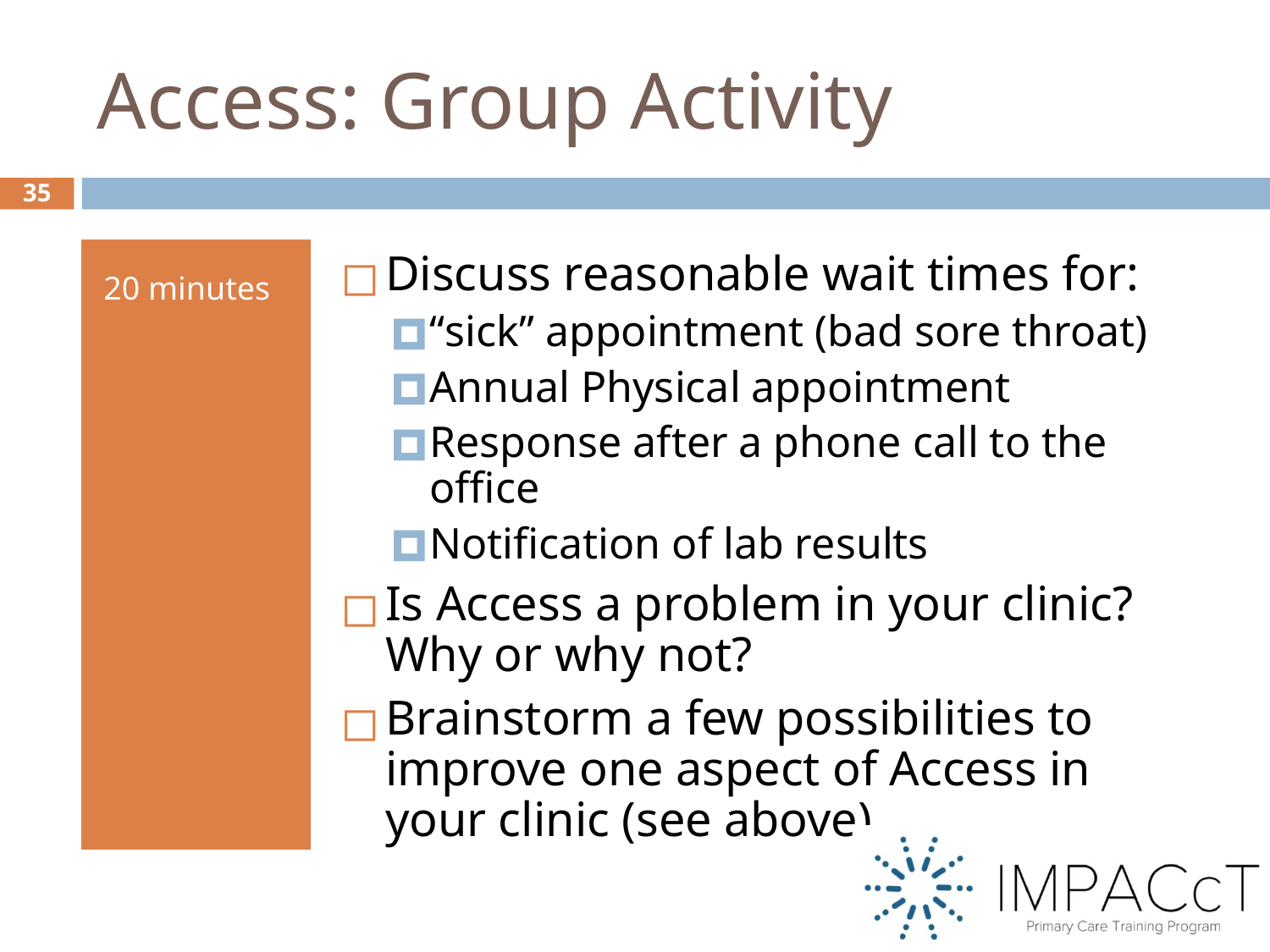

# Access: Group Activity
35
20 minutes
Discuss reasonable wait times for:
“sick” appointment (bad sore throat)
Annual Physical appointment
Response after a phone call to the office
Notification of lab results
Is Access a problem in your clinic? Why or why not?
Brainstorm a few possibilities to improve one aspect of Access in your clinic (see above).

## Slide 36
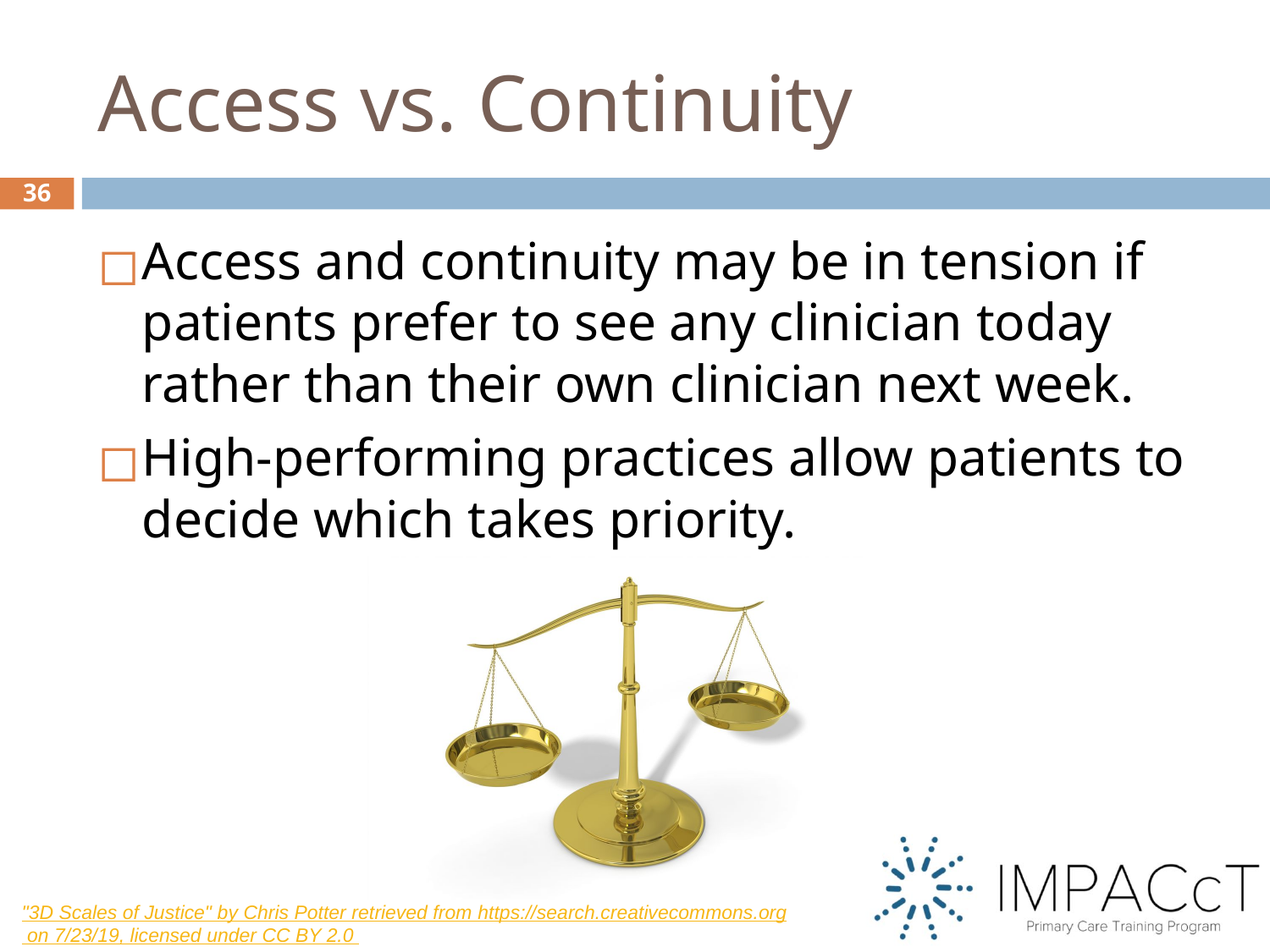

# Access vs. Continuity
36
Access and continuity may be in tension if patients prefer to see any clinician today rather than their own clinician next week.
High-performing practices allow patients to decide which takes priority.
"3D Scales of Justice" by Chris Potter retrieved from https://search.creativecommons.org on 7/23/19, licensed under CC BY 2.0

## Slide 37
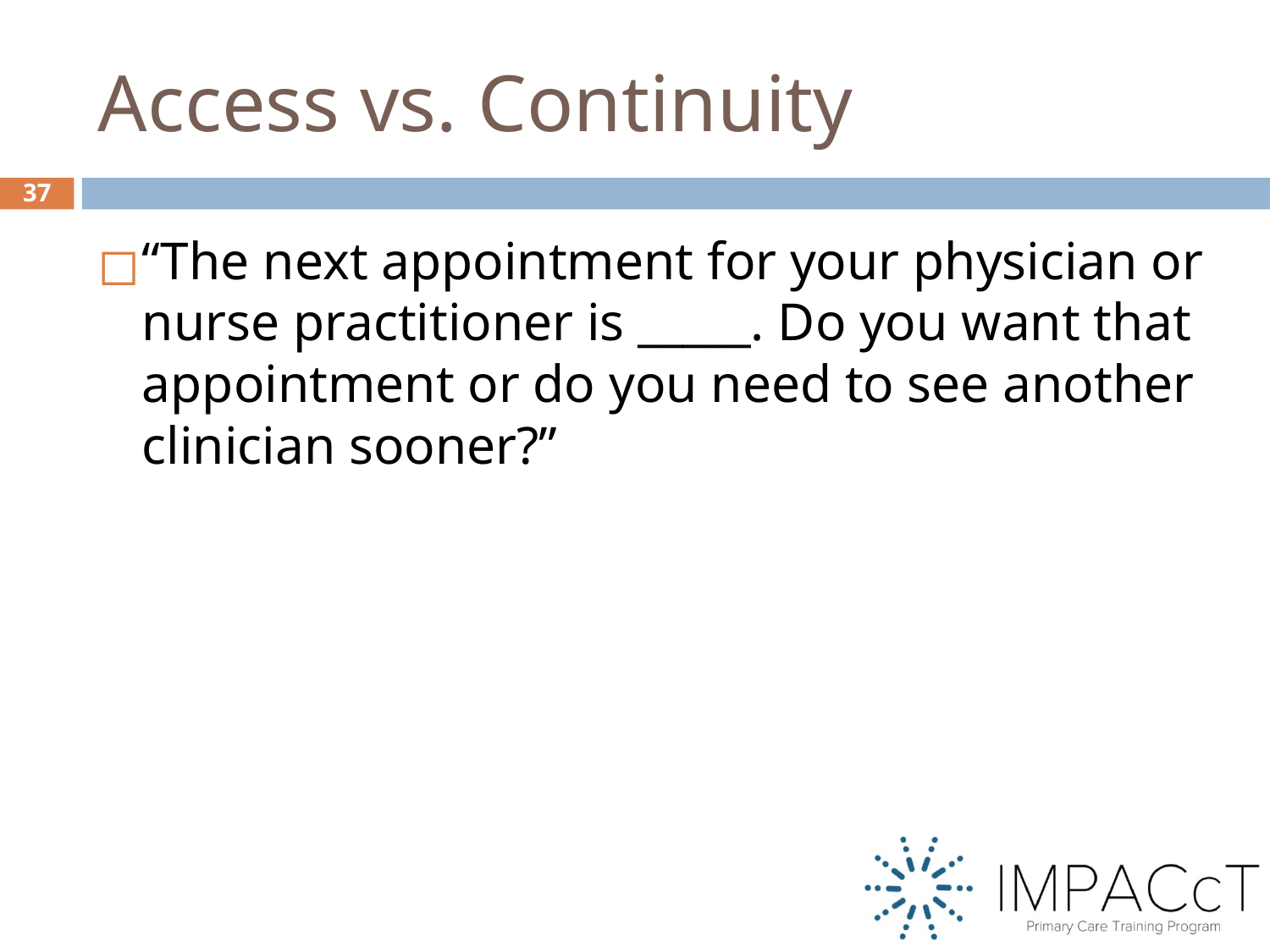

# Access vs. Continuity
37
“The next appointment for your physician or nurse practitioner is _____. Do you want that appointment or do you need to see another clinician sooner?”

## Slide 38
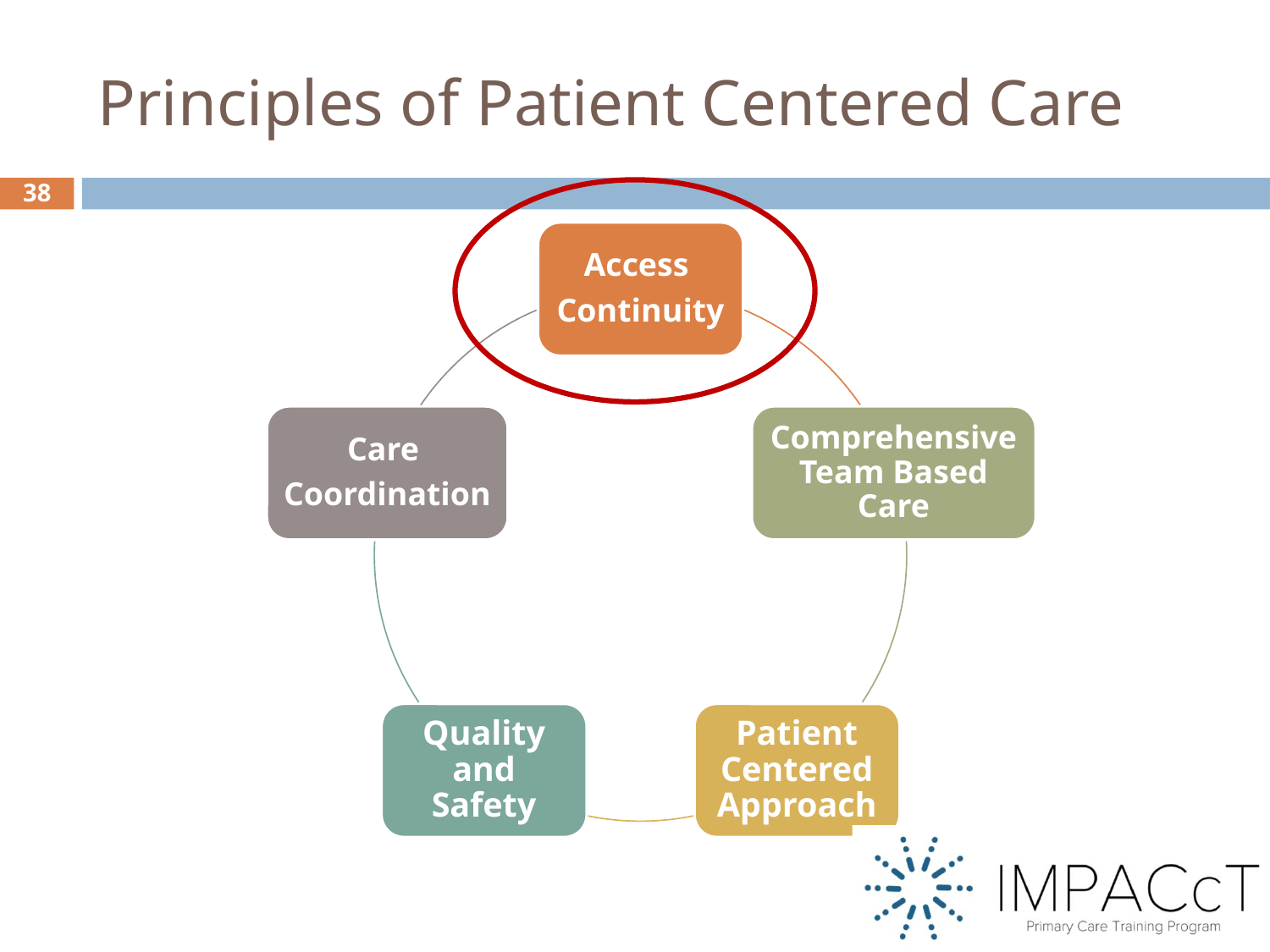

# Principles of Patient Centered Care
38
Access
Continuity
Care
Coordination
Comprehensive Team Based Care
Quality and Safety
Patient Centered Approach
