# Supplementary material for: Access and Continuity: A Multidisciplinary Education Workshop to Teach Patient-Centered Medical Home (PCMH) Principles
Source: MedEdPORTAL. 2020 Oct 7;16:10974. doi: 10.15766/mep_2374-8265.10974 (PMC7549388; doi:10.15766/mep_2374-8265.10974)

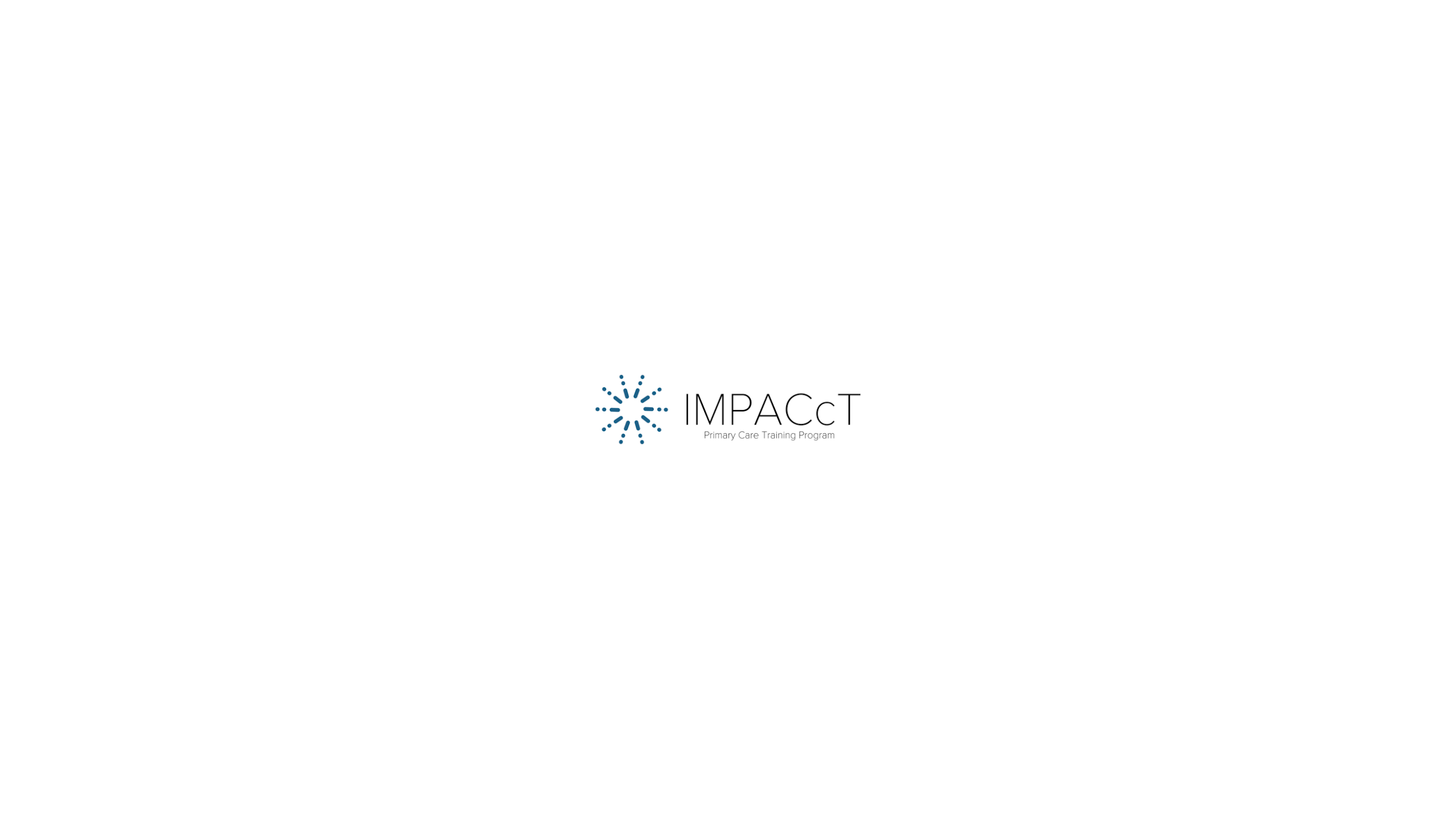


**I am a (check one):**

Medical Resident

Pharmacy Resident

Pharmacy Student

Psychology Extern

Medical Student

PA Student

Faculty/Attending Support Team

Other (_____________)

**Interprofessional Retreat:**

***PCMH Principles: Access and Continuity***

**Program Evaluation**

Write down your “take-home message” from this retreat that you can apply in your clinical practice:

Describe something specific that you learned today and how it might be useful in your work:

Describe something covered today that is ***confusing*** to you or unclear:

General Comments or Feedback [any constructive feedback welcome!]:

AFTER PARTICIPATING IN TODAY’S RETREAT…

***I know more* about the components of a Patient Centered Medical Home .**

0 1 2 3 4 5 6 7 8 9 10

Don’t agree Agree a Agree a moderate Completely

at all a little amount Agree

***I feel more comfortable* with the concepts of access and continuity in a PCMH.**

0 1 2 3 4 5 6 7 8 9 10

Don’t agree Agree a Agree a moderate Completely

at all a little amount Agree

**I think this workshop *made me more prepared* to work within a PCMH model of healthcare delivery.**

0 1 2 3 4 5 6 7 8 9 10

Don’t agree Agree a Agree a moderate Completely

at all a little amount Agree

**In my opinion….**

**This retreat met the educational needs *of all the learners from each profession*.**

0 1 2 3 4 5 6 7 8 9 10

Don’t agree Agree a Agree a moderate Completely

at all a little amount Agree

**This retreat met *my own* educational needs.**

0 1 2 3 4 5 6 7 8 9 10

Don’t agree Agree a Agree a moderate Completely

at all a little amount Agree

**This retreat helped me learn skills that I can apply in the “real world”.**

0 1 2 3 4 5 6 7 8 9 10

Don’t agree Agree a Agree a moderate Completely

at all a little amount Agree
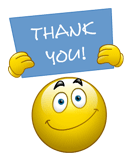

Supplement: Supplementary file 1 — Prework.docxReflective Activity Prompt Slides.pptxReflective Activity Signs for Walls.docxFaculty Guide.docxSlide Presentation.pptxEvaluation Sheet.docx [file mep_2374-8265.10974-s001.zip › F. Evaluation Sheet.docx]
