# Supplementary material for: Access and Continuity: A Multidisciplinary Education Workshop to Teach Patient-Centered Medical Home (PCMH) Principles
Source: MedEdPORTAL. 2020 Oct 7;16:10974. doi: 10.15766/mep_2374-8265.10974 (PMC7549388; doi:10.15766/mep_2374-8265.10974)
Supplement: Supplementary file 1 — Prework.docxReflective Activity Prompt Slides.pptxReflective Activity Signs for Walls.docxFaculty Guide.docxSlide Presentation.pptxEvaluation Sheet.docx [file mep_2374-8265.10974-s001.zip › _Educational_Summary_Report_10974.pdf]

MedEdPORTAL® The AAMC Journal of Teaching and Learning Resources

Original Publication Open Access

Access and Continuity: A Multidisciplinary Education Workshop to Teach Patient-Centered Medical Home (PCMH) Principles

Lauren Block, MD, MPH, Christopher Petersen*, Daniel J. Coletti, PhD, Pratiksha Yalakkishettar, MD, Nancy LaVine, MD

*Corresponding Author: cpetersen1@pride.hofstra.edu

Abstract

Introduction: As more practices move to patient-centered medical home (PCMH) models, future health care professionals must train to work in collaborative settings. We implemented a 3-hour workshop for multidisciplinary trainees on the PCMH principles of access and continuity based on the EFECT framework (eliciting a patient-centered narrative, facilitating an interprofessional team discussion, evaluating the clinical evidence, creating a shared care plan, and tracking outcomes). **Methods:** Participants included internal medicine residents and medical, physician assistant (PA), and clinical psychology students. The workshop incorporated reflective activities identifying patient and provider health care delivery priorities, plus a PCMH presentation and group activities focusing on access and continuity. Evaluations were analyzed qualitatively and quantitatively. **Results:** The workshop had 39 participants (seven physicians, one PA, one educator, one psychologist, three staff, nine residents, one PA student, one psychology extern, and 15 medical students). On a 0-10 Likert scale (0 = *don't agree at all,* 10 = *completely agree*), learners reported higher knowledge of PCMH principles ($M = 8.8$), feeling better prepared for PCMH work ($M = 8.6$), and having obtained real-world skills ($M = 8.3$). Open-ended responses describing the workshop's take-home message included the role of patient-centeredness in clinical redesign, the value of the multidisciplinary team in optimizing access and continuity, and how to use a quality improvement approach for access and continuity. **Discussion:** This workshop increased PCMH-related knowledge and encouraged discussion of professional roles within the team. Learners recognized the benefits of team-based rather than provider-centric approaches to access and continuity.

Keywords
Multidisciplinary Education, Primary Care Training, Patient-Centered Medical Home, Access and Continuity of Care, Experiential Learning, Interdisciplinary Medicine, Interprofessional Education, Quality Improvement/Patient Safety, Internal Medicine

Educational Objectives

By the end of this activity, learners will be able to:

1. Compare and contrast patient- and provider-centered care.
2. Identify the components of a patient-centered medical home (PCMH).
3. Experience the ways patients access care in an ambulatory continuity practice.
4. Brainstorm aspects of an access policy for a resident-based practice, including appointment availability and standards for access to obtain clinical advice.
5. Describe the interpersonal skills needed to work within a PCMH model of health care delivery.
6. Identify ways to enhance continuity of care in a resident practice.
7. Articulate the tension between access and continuity in the delivery of primary care.

Citation:
Block L, Petersen C, Coletti DJ, Yalakkishettar P, LaVine N. Access and continuity: a multidisciplinary education workshop to teach patient-centered medical home (PCMH) principles. *MedEdPORTAL*. 2020;16:10974. https://doi.org/10.15766/mep_2374-8265.10974

Introduction

As more than 12,000 practices[1] across the country have been recognized as patient-centered medical homes (PCMHs), it is important to prepare future health care professionals to work together in PCMH settings.[2] However, best practices describing the best way to train health professionals to work together to provide patient-centered care have yet to be developed.[1,3] Most published reports regarding interprofessional education (IPE) focus on team training and interprofessional interactions, handoff exercises, debriefing tools, and clinical simulations, with minimal literature in *MedEdPORTAL* describing ways to teach PCMH principles through IPE.[4,5]

Copyright © 2020 Block et al. This is an open-access publication distributed under the terms of the Creative Commons Attribution license.

The National Committee for Quality Assurance (NCQA), the accrediting body for PCMHs, has developed core goals for the medical home to (1) enhance access and continuity of care, (2) identify and manage patient populations, (3) plan and manage care, (4) provide self-care and community support, (5) track and coordinate care, and (6) measure and improve performance.[6] These goals align with principles of patient-centered primary care.[7] Entrustable professional activities for trainees have been developed in line with these goals.[3]

Given the importance of patient access and continuity of care in providing high-quality primary care, as well as challenges to continuity in resident practices,[8,9] we developed, implemented, and evaluated a workshop focused on these core PCMH principles. While published literature on PCMH principles has focused on the quality of clinical care delivery and care coordination as core topics for team-based care, this workshop was structured to introduce trainees to the importance of access and continuity as key aspects of patient-centered care and as potential targets for quality improvement projects.

The 3-hour workshop was delivered to members of a primary care training program and resident-led team-based care clinic in 2017. The goals of the workshop were to (1) compare and contrast patient and health care team priorities in care delivery and align these with PCMH tenets, (2) understand the balance required to optimize access and continuity within a residency-based practice, (3) appreciate the interpersonal skills relevant to practice in a PCMH, and (4) identify strategies to improve access and continuity within the practice. The learning process for the workshop was developed based on the EFECT framework for IPE in PCMH, which includes steps of eliciting a patient-centered narrative, facilitating an interprofessional team discussion, evaluating the clinical evidence, creating a shared care plan, and tracking outcomes.[10,11]

Here, we describe our experience designing, delivering, and evaluating this IPE workshop focused on continuity and access as core PCMH principles.

Methods

Participants

The IMPACcT (Improving Patient Access, Care, and Cost through Training) program brings together residents, medical students, psychology externs, physician assistant (PA) students, and pharmacy students to provide multidisciplinary education, career mentoring, and team-based care. Trainees apply through a competitive process overseen by program faculty to join IMPACcT during their program and have protected time to participate in clinical and educational activities. Care is delivered at our core ambulatory training practice, which has been an NCQA-recognized PCMH since 2009. Multidisciplinary education within the IMPACcT program incorporates five half-day workshops yearly, of which this workshop was one.

This workshop was the final program delivered during the 2016-2017 academic year for trainees and faculty from medical, pharmacy, PA, and psychology programs, as well as the medical office assistant and practice coordinator.

Prework

As prework for the workshop, all learners were encouraged to experience the concept of access firsthand by calling their own primary care provider's office to request (a) the next available appointment for a sick visit to address a sore throat and (b) the next available appointment for a physical examination. Trainees were encouraged to note whether these appointments were with their primary provider or a covering provider. Those trainees who did not have a primary provider were encouraged to visit the health system website to examine wait times for care at local emergency departments and urgent care centers. Trainees recorded their experiences to bring to the workshop. Learners also read a brief primer on PCMH principles as prework (Appendix A).

Workshop

The workshop was delivered as a series of four modules.

Module 1: Reflective Activity: Patient- Versus Provider-Centered Care (30 minutes): The first module was a reflective activity comparing and contrasting patient and provider perspectives. Learners were encouraged to consider what was important to them as a patient and as a health care professional and to write their thoughts on orange and green post-it notes, respectively. On the wall were posters listing five PCMH principles: "Access and Continuity," "Coordinated Care," "Comprehensive Team-Based Care," "Quality Improvement and Safety," and "A Patient-Centered Approach." Learners discussed their responses in small groups before placing each note on one of the five posters. Learners discussed where patient and health care professional values aligned and diverged using the posters as a visual representation of priorities. See Appendix B for slides with discussion prompts and Appendix C for posters of PCMH priorities.

Module 2: PCMH Principles Didactic (20 minutes, Appendices D and E, slides 1-9): The second module was a 20-minute didactic presentation on PCMH principles detailing tenets of

Copyright © 2020 Block et al. This is an open-access publication distributed under the terms of the Creative Commons Attribution license.

patient-centered care, an outline of the evidence base for PCMH, and core PCMH principles. Specific focus was placed on the principles of access and continuity, including assessment and monitoring of these principles.

Module 3: Continuity (40 minutes, Appendices D and E, slides 10-22): The third module was a group activity focused on continuity. Unique challenges to continuity in resident practices were described using local data as well as case studies from other residency programs based on the Greater New York Hospital Association toolkit.[12] Continuity data from the IMPACcT practice and a more traditional resident clinic in our health system were reviewed. Trainees then discussed the definition of continuity in a team-based practice and developed an idea to improve continuity in the IMPACcT practice. Small groups chose a representative to report their ideas to the larger group, and presenters facilitated a discussion of potential quality initiatives for our training practice. Please see Appendix D for the facilitator's guide and Appendix E for large-group slides.

Module 4: Access (40 minutes, Appendices D and E, slides 23-34): The fourth module included group activity focused on access. Approaches to measuring access and innovative approaches to improving access were discussed using local data and published case studies.[11] Drawing on learners' assigned prework with their own primary care providers, we discussed learners' access experiences. Small groups were asked to consider reasonable wait times for different patient access types: sick visit, response to a phone call, and notification of lab results. These groups then brainstormed ways to improve one aspect of access in the IMPACcT practice using a team-based care model and reported these to the larger group. Finally, trainees discussed potential tension and trade-offs between access and continuity.

Evaluation (15 Minutes, Appendix F)

All trainees and faculty were asked to complete an evaluation that included four quantitative questions on the value of the workshop as well as an assessment of knowledge gained (Appendix F). Open-ended questions included the following:

1. Write down your take-home message to apply in future IPE clinical situations.
2. Describe a skill you learned today.
3. Describe something that is still confusing to you.

Likert-scale responses were examined descriptively. Open-ended responses from trainees and faculty were analyzed qualitatively using a step-by-step thematic analysis performed by two researchers (Christopher Petersen and Lauren Block) reading through each student's postsession evaluation comments. Researchers reviewed evaluations multiple times to familiarize themselves with the comments. Researchers generated initial codes and then grouped codes into themes, which were pooled to the point of saturation. Analyst triangulation was conducted by having a third researcher (Nancy LaVine) review the final themes to ensure consistency with session goals and learning objectives.[13] This evaluation was considered a quality improvement activity by our institution's institutional review board.

Results

This workshop included 39 participants: nine second- and third-year residents, one second-year PA student, one psychology extern, 15 first- through third-year medical students, 10 multidisciplinary faculty (seven from medicine, one PA, one graduate medical education, and one psychology), and one administrative and two clinical staff members. We obtained 31 end-of-retreat evaluations (79%) from this group.

Retreat evaluations (see Figure) revealed that subsequent to participation, learners reported increased knowledge of PCMH principles ($M = 8.8$ on a 0-10 Likert scale where 0 = *not at all,* 3 = *somewhat,* 7 = *mostly,* and 10 = *completely*), felt better prepared to work in a PCMH (8.6), and had acquired real-world skills ($M = 8.3$). Learners also felt the workshop met the needs of the diverse range of attendees ($M = 7.7$).

Open-ended questions from session evaluations generated several themes: the importance of patient-centeredness in clinical redesign, the value of the team in optimizing access and quality, and ways to use quality improvement to improve access and continuity (see Table). The skills learners reported acquiring included ways to use the electronic medical record to improve access and continuity through empanelment and patient navigation, strategies to address the tension between access and continuity, quality improvement approaches to improve the patient experience, and ways to optimize between-visit care. Learners expressed interest in learning more about the process of pursuing PCMH certification and whether access and continuity experiences in the team-based practice were generalizable to other primary care practices.

Discussion

This multidisciplinary workshop increased learner knowledge of PCMH principles and prepared learners to work collaboratively to optimize access and continuity. Through the reflective activity, learners were able to reflect on the experience as a patient,

Copyright © 2020 Block et al. This is an open-access publication distributed under the terms of the Creative Commons Attribution license.

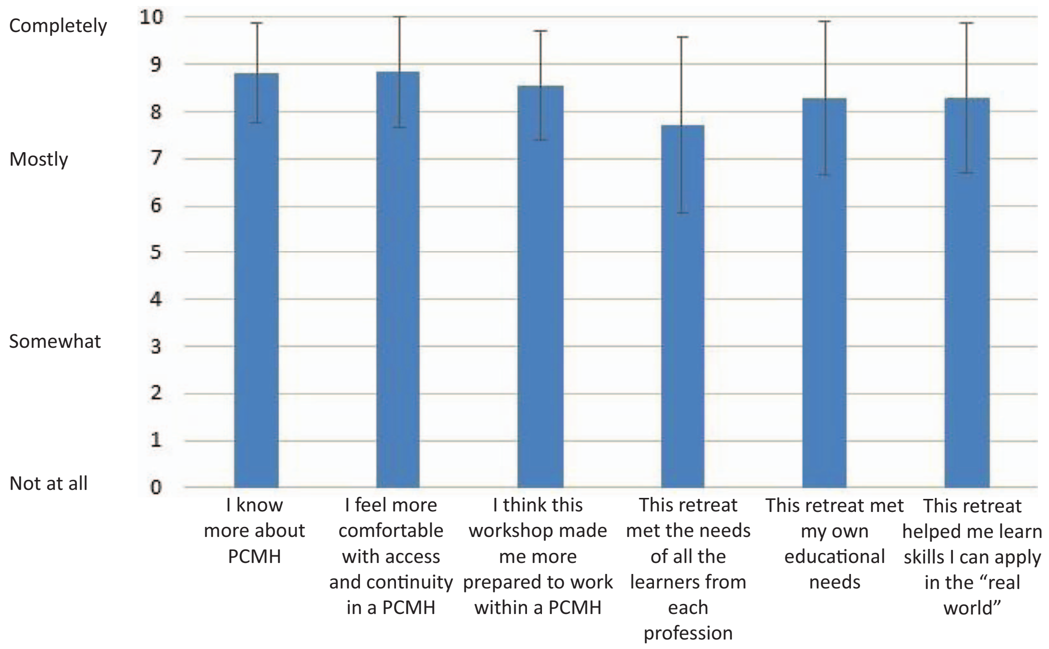

Figure. Quantitative evaluation data. Abbreviation: PCMH, patient-centered medical home. Error bars indicate one standard deviation.

highlighting the importance of access and continuity in patient-centered care. The didactic presentation and group activities focused on brainstorming ways to improve access and continuity in a multidisciplinary training practice, allowing learners to take a big-picture approach to clinical care design, a role they did not regularly play in training clinics. Furthermore, the reflective and small-group activities helped to foster skill building and multidisciplinary relationships through understanding the trade-offs between continuity and access inherent in proposed quality improvement programming.

A key strength of this workshop was how its inclusive and multidisciplinary learning environment facilitated the conception of continuity as team based rather than provider specific. For example, the specific roles of identifying preventive health care and vaccination needs were assigned to the PA and pharmacy students to maximize participation in the daily huddle.

Table. Qualitative Retreat Evaluation Data (*N* = 31)

Evaluation Tool Criteria	Most Common Responses
Write down your take-home message.	"I learned more about PCMH."<br>"Patient centeredness is a key to clinical design: Think like a patient."<br>"Value of team in optimizing quality improvement to access & continuity."
Describe a skill learned today.	"Consider patients concerns when interacting with phone call."<br>"Quality improvement can improve patient experience as well as providers'."<br>"Tension exists between access and continuity."
Describe something that is still confusing to you.	"Which practices are PCMH certified?"<br>"Are these ideas theoretical vs. practical, and how useful outside of IMPACcT?"

Abbreviations: IMPACcT: Improving Patient Access, Care, and Cost through Training; PCMH, patient-centered medical home.

Prior descriptions of PCMH curricula have focused on care coordination and team-based care as core PCMH principles.[5,6] The existing literature predominantly concentrates on the collaborative practices regarding management of chronic disease and transitional care centered around the nurse practitioner, clinical pharmacist, and nurse care coordinators.[5] In contrast, the IMPACcT program utilizes residents, medical students, psychology externs, PA students, pharmacy students, attending physicians, clinical psychologists, and pharmacists to provide multidisciplinary care. Most significantly, this workshop focused on access and continuity as core aspects of patient-centered care in a PCMH practice, rather than coordinating care related to specific patient groups. The workshop capitalized on the knowledge of the multidisciplinary team in understanding the importance of access and continuity to both patients and providers. Learners were able to recognize how changes to health care delivery systems impact continuity and access and could view access and continuity as potential targets for quality improvement projects. Through the novel multidisciplinary structure of the workshop, learners were exposed to the underlying importance of team-based care and of utilizing team-based care coordination to address the other tenets of a PCMH.

Copyright © 2020 Block et al. This is an open-access publication distributed under the terms of the Creative Commons Attribution license.

Although the project benefited from the input of diverse multidisciplinary faculty in a large health care institution, this report presents findings from a single workshop delivered at one institution. Because this workshop focused on access and continuity, other PCMH principles received less focus. Our evaluation concentrated on trainee attitudes and reported knowledge, rather than on skill assessment. While our workshop included trainees from four professions, our university does not house an undergraduate nursing or social work program; thus, these important health care professionals were not included in this iteration of the workshop. While the workshop did focus on a multidisciplinary clinic approach to care, lessons in PCMH principles remain applicable to any resident-based training clinic.

Subsequent work has included annual multidisciplinary workshops focused on other PCMH topics, including transitions of care. Through ongoing quality improvement projects, trainees work together on concrete steps to improve access and continuity in the IMPACcT practice. Next steps will include inviting all members of the PCMH, including nurses and social workers, to join in these initiatives. Training on PCMH principles helps ensure all health care providers and trainees recognize that they are part of a medical home working towards common goals of patient-centered, coordinated, team-based care. By understanding this context, trainees, staff, and faculty can work to optimize processes of care including access and continuity.

Appendices

A. Prework.docx

B. Reflective Activity Prompt Slides.pptx

C. Reflective Activity Signs for Walls.docx

D. Faculty Guide.docx

E. Slide Presentation.pptx

F. Evaluation Sheet.docx

All appendices are peer reviewed as integral parts of the Original Publication.

Lauren Block, MD, MPH: Associate Professor, Department of Medicine, Donald and Barbara Zucker School of Medicine at Hofstra/Northwell

Christopher Petersen: Medical Student, Donald and Barbara Zucker School of Medicine at Hofstra/Northwell

Daniel J. Coletti, PhD: Assistant Professor, Department of Medicine, Donald and Barbara Zucker School of Medicine at Hofstra/Northwell

Pratiksha Yalakkishettar, MD: Resident, Department of Family Medicine, University of Massachusetts Medical School

Nancy LaVine, MD: Assistant Professor, Department of Medicine, Donald and Barbara Zucker School of Medicine at Hofstra/Northwell

Disclosures

None to report.

Funding/Support

A portion of our project (IMPACcT: Improving Patient Access, Care, and Cost through Training) is funded by the Health Resources and Services Administration of the U.S. Department of Health and Human Services through a Primary Care Training Enhancement (PCTE) Grant (#TOBHP28558).

Ethical Approval

Reported as not applicable.

Disclaimer

The contents of this publication are those of the authors and do not necessarily represent the official views of, or an endorsement by, the Health Resources and Services Administration, the U.S. Department of Health and Human Services, or the U.S. Government.

References

1. Patient-Centered Medical Home (PCMH) Recognition: overview of PCMH. *National Committee for Quality Assurance.* Accessed August 22, 2018. http://www.ncqa.org/programs/recognition/practices/patient-centered-medical-home-pcmh
2. Reynolds PP, Klink K, Gilman S, et al. The patient-centered medical home: preparation of the workforce, more questions than answers. *J Gen Intern Med*. 2015;30(7):1013-1017. https://doi.org/10.1007/s11606-015-3229-2
3. Chang A, Bowen JL, Buranosky RA, et al. Transforming primary care training—patient-centered medical home entrustable professional activities for internal medicine residents. *J Gen Intern Med*. 2013;28(6):801-809. https://doi.org/10.1007/s11606-012-2193-3
4. Karpa K, Messmer J, Jiang E, Leong SL. Demonstrating team-based care in primary care environments via video modules and discussion. *MedEdPORTAL*. 2015;11:10116. https://doi.org/10.15766/mep_2374-8265.10116
5. Danielson J, Johnson G, Davidson H. Patient-centered medical home (PCMH) simulation: planning for coordinated care visits in the primary care clinic. *MedEdPORTAL*. 2014;10:9795. https://doi.org/10.15766/mep_2374-8265.9795
6. Higgins TC, Schottenfeld L, Crosson J. *Primary Care Practice Facilitation Curriculum—Module 25: The Patient-Centered Medical Home: Principles and Recognition Processes*. Agency for Healthcare Research and Quality; 2015. AHRQ publication no.

Copyright © 2020 Block et al. This is an open-access publication distributed under the terms of the Creative Commons Attribution license.

15-0060-EF. Accessed August 22, 2018. https://pcmh.ahrq.gov/sites/default/files/attachments/pcpf-module-25-pcmh-principles.pdf

7. Davis K, Schoenbaum SC, Audet AM. A 2020 vision of patient-centered primary care. *J Gen Intern Med*. 2005;20(10):953-957. https://doi.org/10.1111/j.1525-1497.2005.0178.x
8. Bodenheimer T, Ghorob A, Willard-Grace R, Grumbach K. The 10 building blocks of high-performing primary care. *Ann Fam Med*. 2014;12(2):166-171. https://doi.org/10.1370/afm.1616
9. Bowen JL, Hirsh D, Aagaard E, et al. Advancing educational continuity in primary care residencies: an opportunity for patient-centered medical homes. *Acad Med*. 2015;90(5):587-593. https://doi.org/10.1097/acm.0000000000000589
10. Kolb DA, Boyatzis RE, Mainemelis C. Experiential learning theory: previous research and new directions. In: Sternberg RJ, Zhang LF, eds. *Perspectives on Thinking, Learning, and Cognitive Styles*. Taylor & Francis Group; 2000:227-247.
11. Bitton A, Pereira AG, Smith CS, Babbott SF, Bowen JL; Society of General Internal Medicine Patient-Centered Medical Home Education Summit. The EFECT framework for interprofessional education in the patient centered medical home. *Healthc (Amst)*. 2013;1(3-4):63-68. https://doi.org/10.1016/j.hjdsi.2013.08.003
12. Greater New York Hospital Association. *Redesigning the Teaching Clinic: A Toolkit for Improving Care Coordination and Resident Learning*. Greater New York Hospital Association; 2012.
13. Patton MQ. Enhancing the quality and credibility of qualitative analysis. *Health Serv Res*. 1999;34(5)(pt 2):1189-1208.

Received: January 31, 2020
Accepted: March 26, 2020
Published: October 7, 2020

Copyright © 2020 Block et al. This is an open-access publication distributed under the terms of the Creative Commons Attribution license.
